# Phage Therapy in Gastrointestinal Diseases: Current Status and Challenges

**DOI:** 10.3390/ijms27083662

**Published:** 2026-04-20

**Authors:** Shaokun Zhang, Ying Zhang

**Affiliations:** School of Medicine, Southeast University, Nanjing 211189, China; 213220777@seu.edu.cn

**Keywords:** phages, gastrointestinal diseases, gut microecology, clinical translation

## Abstract

A phage is a virus that targets bacteria with high precision. While phage therapy provides a targeted alternative to broad-spectrum antibiotics, it is not completely free from the challenges of antimicrobial resistance, as phages can facilitate the horizontal transfer of resistance genes through transduction and promote the growth of phage-resistant strains. Nonetheless, within the One Health framework, the strategic use of phages remains a vital and promising tool for addressing the global antimicrobial resistance crisis. This paper reviews current research on phage therapy for gastrointestinal diseases such as cirrhosis, enteritis, and Helicobacter pylori infection. It also details how phages help regulate gut microecological balance and discusses how phage dysbiosis can lead to innate immune dysfunction and worsen conditions like inflammatory bowel disease. The review summarizes both the therapeutic potential and limitations observed in clinical trials and fundamental studies. Transitioning from laboratory research to clinical practice is hindered by multiple complex challenges, including the stomach’s extreme acidity, physical entrapment by the intestinal mucus layer, the rapid co-evolution of bacterial resistance, and ecological risks associated with temperate phages. To overcome challenges like gastrointestinal barrier tolerance and address ethical, technical, and practical hurdles for clinical use, the paper outlines treatment strategies for specific conditions and highlights future directions, providing guidance for employing phages in digestive system disease management. These future innovations focus on integrating artificial intelligence-driven precision matching, advanced bioengineering for durable delivery systems, and multimodal combination therapies to safely modulate the intestinal microecology.

## 1. Introduction

Gastrointestinal (GI) diseases, encompassing a wide spectrum of conditions such as cirrhosis-associated gastrointestinal infections [1], *Helicobacter pylori* infections [2], and inflammatory bowel disease (IBD) [2], impose a profound and escalating burden on global healthcare systems. Historically, broad-spectrum antibiotics have served as the cornerstone of treatment for these pathologies; however, this paradigm is increasingly failing [1]. The widespread overuse of traditional antibiotics has actively driven the global emergence of antimicrobial resistance (AMR), creating a formidable clinical challenge. Furthermore, antibiotics act as non-selective agents that inflict severe collateral damage upon the delicate balance of the intestinal microecosystem. This disruption frequently induces dysbiosis and secondary complications, such as dyspepsia and diarrhea, ultimately contributing to poor treatment outcomes and high relapse rates among patients [3]. Consequently, there is an urgent clinical mandate to develop novel, safe, and highly efficacious targeted therapeutics that bypass the mechanisms of conventional drug resistance.

Bacteriophages offer a compelling alternative that directly addresses the limitations of traditional antimicrobial agents. As naturally occurring, non-cellular microorganisms composed primarily of a nucleic acid genome and a protein capsid, phages function as obligate viral predators of bacteria. Unlike broad-spectrum antibiotics, phages exhibit extreme strain-level specificity. They are capable of precisely targeting, infecting, and lysing pathogenic bacteria without inflicting collateral damage upon the beneficial commensal microflora of the human body. Because their bactericidal mechanisms differ fundamentally from those of chemical antibiotics, phage therapy effectively avoids the widespread collateral damage associated with traditional drugs [4]. However, it is important to acknowledge that phages are intertwined with the AMR cycle; temperate phages can facilitate the spread of antibiotic resistance genes through transduction, and lytic phages exert selective pressure that drives bacterial resistance evolution. Despite these challenges, from a One Health perspective, phage therapy is increasingly recognized as an essential component of a multifaceted strategy to reduce the overall burden of the global AMR crisis.

Beyond their potential as exogenous therapeutics, bacteriophages are deeply integrated into the human gastrointestinal tract. They exist in complex, dynamic relationships with the gut microbiota, operating through both lytic and lysogenic life cycles to maintain the stability and diversity of the digestive microecosystem [5]. However, harnessing these ubiquitous viruses for clinical application requires navigating significant biological hurdles. Bacteria, as co-evolving organisms, can rapidly develop specific phage resistance [5], and the harsh physiological environments of the GI tract pose significant challenges to the survival and delivery of active phage particles. Furthermore, in certain cases, phages may exacerbate the disease.

This paper critically examines the current landscape of phage therapy across various gastrointestinal diseases, integrating recent findings from both preclinical studies and clinical trials. By carefully analyzing the therapeutic potential alongside the significant technical, ethical, and practical challenges of phage application, this review aims to provide a thorough framework. Ultimately, it highlights future technological and clinical directions—such as advanced delivery systems and combined therapies—offering essential guidance for translating precision phage therapy into routine clinical practice for digestive system disorders.

## 2. The Interplay Between the Biological Characteristics of Bacteriophages and the Gastrointestinal Microecosystem

### 2.1. Fundamental Biological Characteristics of Bacteriophages

To fully harness the therapeutic potential of the bacteriophages outlined above, it is essential first to understand their fundamental biological characteristics and intricate ecological roles in the human gut. Phages are non-cellular microorganisms, with the most basic structure being a nucleic acid genome and a protein capsid [5]. Based on metagenomic analysis, the phageome appears to be primarily composed of double-stranded DNA viruses. Based on their life cycle, bacteriophages are divided into two types: one is the lytic phage, which can directly kill the target bacteria, and the other is the temperate phage, which can integrate into the bacterial genome [5]. The relationship between the phages could be not only parasitic but also symbiotic. After infecting bacteria, lytic phages can quickly reproduce and destroy host cells to release progeny viruses. In the process of killing bacteria, it goes through adsorption, penetration, replication, assembly, and release. It will not damage normal human cells but will directly kill the target bacteria, which is sufficient to demonstrate its specificity. Conversely, temperate phages can integrate their own genes into bacterial chromosomes or exist as plasmids, which can be passed on to daughter cells during replication. After integration, the phage can remain dormant for a long time. If it encounters a variety of signals, including oxidative stress and DNA damage, it will switch to the lytic cycle. To clarify these distinct life cycles and their therapeutic implications, Table 1 compares the core attributes of lytic and temperate phages [6].

### 2.2. Distribution Patterns of Bacteriophages in the Human Gastrointestinal Tract

Bacteriophages exhibit significant spatial heterogeneity that governs the uneven colonization patterns across niches [16]. Due to the vast differences between the oral cavity and the large intestine in terms of oxygen gradients, pH levels, salivary flushing shear forces, and nutrient substrates, the oral phageome and the gut phageome differ significantly in community composition and life history strategies. Oral phages are typically exposed to external environmental factors more frequently, and therefore often exist as temperate phages, maintaining long-term equilibrium with their hosts [17].

Gastric acid limits microbial survival, yet phages persist. The significant discovery of Helicobacter pylori suggests that people’s understanding of the gastric environment requires revision [18]. Furthermore, although the stomach’s pH appears too low, some phages can persist in the gastric mucosa for extended periods [19]. Because phages have evolved strategies such as predation and lysogenic conversion, and prophages in the stomach exhibited more conserved genetic structures, this suggests adaptation to this specific niche.

Moreover, phage numbers increase from the duodenum to the colon, a pattern linked to bacterial density [15]. Transmission electron microscopy reveals that most phages in human feces belong to the class *Caudoviricetes*, displaying myovirus, podovirus, and siphovirus morphotypes [20]. Based on metagenomic analysis, the phageome appears to be primarily composed of double-stranded DNA viruses. The most abundant virus reflects the dominance of crAss-like phages (Crassvirales), which the data indicate are present in more than 70% of the global population [14]. Furthermore, crAss-like phage accounts for up to 99% of the phage community in some individuals [15]. Thus, crAss-like phage dominates the human phageome globally. Table 2 outlines the general characteristics, nucleic acid types, and typical bacterial targets of these key phage groups.

In 2021, the Gamarillo-Guerrero Institute used 28,060 human gut metagenomes and 2898 bacterial genomes from around the world to construct a database containing 142,809 phage types, more than half of which are new viruses. This study further clarifies the diversity of phages in the human gut and indicates that the crAss-like phage clade is the most common [21].

In recent research, the quantitative relationship between phages and bacteria has been analyzed in depth, yielding a series of quantitative data. Numerous studies have found that the ratio of phage particles to bacterial cells in the human body is 1:100, whereas the ratio of phage genome to bacterial genome is 4:1 [8]. This conclusion seems paradoxical, but it also shows that phages in the human body exist not as free particles but as prophages integrated into bacterial chromosomes. It is estimated that half of the bacteria in the human body can carry at least one prophage, and the proportion of prophages in bacterial genomes can reach 1–5% [15].

**Table 2:** Morphological and genomic characteristics of major bacteriophage groups.

Phage Group/Morphotype	Morphology/ Structure	Typical Size Range	Nucleic Acid Type	Examples of Target Bacteria	References
***Caudoviricetes (Myovirus)***	Icosahedral head with a long, contractile tail	Head: ~50–140 nm; Tail: ~80–120 nm	dsDNA	*Escherichia coli, Shigella*	[20]
***Caudoviricetes (Siphovirus)***	Icosahedral head with a long, non-contractile tail	Head: ~50–70 nm; Tail: up to 500 nm	dsDNA	*Lactococcus,* *Helicobacter pylori*	[20,22]
***Caudoviricetes (Podovirus)***	Icosahedral head with a short, non-contractile tail	Head: ~50–70 nm; Tail: ~10–20 nm	dsDNA	*Klebsiella pneumoniae,* *Salmonella* spp.	[20,23]
**crAss-like phages** ***(Crassvirales)***	Icosahedral head with a short tail	Head: ~80 nm	dsDNA	*Bacteroides* spp.	[15,21]
**Filamentous Phages** ***(Inoviridae)***	Long, flexible filament lacking a tail structure	Length: ~700–3000 nm; Width: ~6–8 nm	ssDNA	*Escherichia coli, Vibrio cholerae*	[6]

### 2.3. The Symbiotic Relationship Between Bacteriophages and the Gut Microbiota and Its Molecular Mechanisms

The relationship between the phages could not only be parasitic, but also symbiotic. Phages have two lifestyles in the intestinal ecosystem: lytic and lysogenic. After infecting bacteria, lytic phages can quickly reproduce and destroy host cells to release progeny viruses. Temperate phages can integrate their own genes into bacterial chromosomes or exist as plasmids, which can be passed on to daughter cells during replication [7].

At the initial stage of the research, it was believed that the phage in the human body was in a state of lysis, but quantitative analysis showed that it was temperate, regardless of whether the genome was in a state of lysis or temperance. In the estimation, the ratio of viral particles to bacterial cells is taken into account, and it is found that phage-induced lysis is relatively low, occurring only 0.001 to 0.01 times per day [8]. The induction rate is relatively low, so it does not impose a significant fitness cost on bacteria, and phages and bacteria can coexist for a long time, which is conducive to the stability of the intestinal ecosystem.

After the phage infects the bacterium, it can decide whether to enter the lytic cycle or remain in lysogeny, depending on the bacterium’s growth environment and physiological state. The establishment of lysogeny requires the support of a series of complex molecular regulatory mechanisms. If the bacteria are in a nutrient-deficient state, lysogeny is more likely to be established. After integration, the phage can remain dormant for a long time. If it encounters a variety of signals, including oxidative stress and DNA damage, it will switch to the lytic cycle [15].

At the molecular level, the symbiosis between phages and bacteria is realized in various ways. Temperate phages can insert their own genome into specific sites (attB/attP) in the bacterial chromosome via an integrase, thereby achieving vertical transmission [24]. The integration of the two can not only prevent the phage from decaying in the environment but also improve the bacteria’s adaptability.

The presence of prophages can make host bacteria more adaptable to their environment, and there are many ways to achieve this. First, the presence of “auxiliary genes” can play an important role in bacterial development, including nutrient acquisition, enhanced defence, and toxin production. Although these genes have no direct impact on phage life cycles, they can enhance the competitiveness of lysogens [9]. Second, the integration of prophages could affect bacterial genes, and their metabolism will change due to gene silencing [25]. Third, the presence of prophages can enhance bacterial immunity to phages [10]. Fourth, some prophages can generate diverse retroelements, which can alter bacterial genes and provide a basis for bacterial genetic diversity [26].

It should be noted that the lysogenic state is not static. Some scholars have pointed out that temperate phages can infect *Bacteroides* spp. and that their genome diversity is relatively high, significantly higher than that of other lytic phages. The genome has been detected in a variety of species, indicating that the host range of this phage is relatively broad [27]. The host has strong adaptability, which can promote gene exchange between different bacterial lineages and facilitate dissemination [28].

Phages can interact with bacteria, and the three parties can form a network relationship through the intestinal epithelium [29]. During phage–epithelium interaction, the Bacteriophage Adherence to Mucus (BAM) model is formed [26], enabling the phage to interact with epithelial cells through the mucus layer. Contact between phage and epithelium can promote the transfer of nucleic acid and proteins, activate cellular signalling cascades that promote nutrient secretion and glycosylation [27], and promote the symbiosis of specific bacteria [30]. Generally speaking, the feedback loop between phages and bacteria can offer a new approach for analyzing the microecosystem.

### 2.4. Mechanisms of Phage-Mediated Regulation of Gut Microecological Homeostasis

Bacteriophages can selectively lyse specific bacteria, which is the most direct way to adjust the microbial community structure. After infecting the host, lytic phages go through four stages: adsorption, genome injection, replication and assembly, and lysis and release [11], which can quickly reduce the population density of target bacteria. This is an effective way to maintain microbial diversity and has a strong ecological significance. In the “kill-the-winner” model, when a certain type of bacteria multiplies rapidly, the corresponding bacteriophages will also increase in abundance, and the population will be effectively lysed, thereby creating more ecological niches for other bacteria and preventing the monopolization of resources by a single type of bacteria [11].

Phage-mediated gene transfer significantly contributes to the spread of antibiotic resistance genes (ARGs). Many studies have reported that ARGs are more diverse in human fecal samples, including beta-lactamase and quinolone resistance genes [12]. These resistance genes can be transferred between bacteria, and some phages can transfer them across species such as *Enterococcus, Staphylococcus*, and *Streptococcus*, etc. [31]. The use of antibiotics will impose strong selective pressure, which will drive the spread of ARGs through phage transduction [13,32].

Phages also carry a set of auxiliary metabolic genes that can play an important role in bacterial metabolism. The latest research shows that phages can encode *α-1,2-fucosyltransferase (futC)* genes, which encode *α-1,2-fucosyltransferase (futC)*, which synthesizes 2′-fucosyllactose (2′-FL) [33]. 2′-FL is an important component of human milk and can enhance intestinal barrier function. Human gut phage-derived genes encode specific metabolic genes (such as *futC*) that direct the biosynthesis of 2′-FL both in vitro and in vivo. The accumulation of 2′-FL not only enhances mucosal barrier integrity but also, by modulating the gut microbiota, promotes the release of secretory IgA and the development of intraepithelial CD4+CD8αα+ T cells, thereby significantly alleviating dextran sulphate sodium (DSS)-induced colitis in mouse models [34]. This conclusion shows that phages can encode functional genes that regulate host immune function and improve intestinal barrier function, thereby promoting intestinal homeostasis.

Phages can not only interfere with the host’s immunity but also interact with the immune system. Some scholars have pointed out that the presence of phage particles can reduce reactive oxygen species(ROS) levels [35], block T cell activation, and inhibit the NF-κB signalling pathway, thereby playing an anti-inflammatory role [36]. Furthermore, immunosuppression induced by phage administration in the gut has been shown to concurrently abrogate humoral and cellular immunity [37].

**Figure 1:**
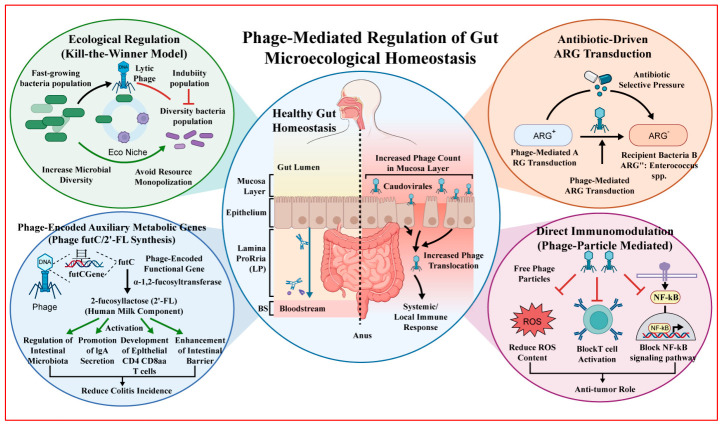
Phage-mediated regulation of gut microecological homeostasis. The figure illustrates four principal mechanisms through which phages interact with the gut ecosystem: (1) Ecological Regulation (**Top Left**): Lytic phages maintain bacterial diversity via the “kill-the-winner” model, preventing resource monopolization by rapidly proliferating strains. (2) ARG Transduction (**Top Right**): Under antibiotic pressure, phages can mediate horizontal gene transfer, inadvertently spreading antimicrobial resistance genes (ARGs). (3) Metabolic Gene Expression (**Bottom Left**): Phages encode auxiliary metabolic genes (e.g., *futC*) that promote 2′-fucosyllactose (2′-FL) synthesis, enhancing the intestinal barrier and secretory IgA production. (4) Immunomodulation (**Bottom Right**): Free phage particles can directly interact with the host immune system to reduce reactive oxygen species (ROS) and block NF-κB inflammatory signalling pathways.

In a healthy body, the number of phages in the blood is relatively small, which can break through the intestinal epithelial barrier. When inflammatory bowel diseases (IBD) occur, the number of phages in the mucosal layer increases [38], the intestinal epithelial barrier is damaged, and more phages can enter the bloodstream and lamina propria, thereby triggering systemic or local immune responses [33]. This conclusion indicates a relationship between phage translocation and intestinal inflammation, as shown in Figure 1.

## 3. Current State of Phage Therapy Research in Various Gastrointestinal Diseases

### 3.1. Bacterial Infections of the Gastrointestinal Tract

While the human gastrointestinal tract is vulnerable to many bacterial pathogens, this review focuses on Clostridioides difficile and Helicobacter pylori infections due to the unique clinical scenarios they present. *C. difficile* infection exemplifies antibiotic-induced dysbiosis, where traditional broad-spectrum treatments often cause high recurrence rates [35]; therefore, phage therapy provides a vital microbiota-preserving alternative [39]. *H. pylori* is deeply embedded in the highly acidic environment of the stomach and poses a significant global challenge due to rapidly increasing multidrug resistance [40,41]. These two pathogens demonstrate both the great potential of microbiome-targeted therapies and the significant biophysical obstacles (such as sporulation [42] and gastric acidity [43]) that phage treatments must overcome, emphasizing the need for detailed, focused analysis.

#### 3.1.1. Clostridium Difficile Infection

In the process of preventing and treating bacterial gastrointestinal diseases, it is necessary to pay attention to the problems caused by *Clostridium difficile* infection, which is a common nosocomial infection in clinical practice [44]. The main challenge in treating antibiotic diarrhea is the high recurrence rate: up to 25% after initial antibiotic therapy, and as high as 75% in patients with multiple treatments [35]. In addition, patients with other diseases, such as immunodeficiency and chronic kidney disease, are more likely to develop antibiotic-associated diarrhea [45]. In this case, phage therapy has gained attention for its dual role in supporting the balance of gut microbiota and destroying pathogens [39].

In the preclinical study, the researchers used the clinical isolate of *C. difficile* to construct a biofilm model. Viable cell counting, scanning electron microscopy, and confocal imaging showed that the four-phage cocktail was rationally optimized and completely eliminated biofilm formation when it was used in the preventive stage (phage was given before bacterial inoculation). Although the cocktail does not completely eliminate the spores, it forms obvious plaques and reduces the number of cells significantly [42]. In the larval model, the phage cocktail was given orally before the bacterial challenge, and the survival rate reached 100%. By comparison, the survival rate of therapeutic administration was only 30%, but the survival rate was increased to 60% by increasing the dose, which showed that it was necessary to use multiple doses [42].

In order to eliminate the risk of lysogeny, the researchers deleted the lysogeny-related genes in the temperate phage and used the CRISPR-Cas system to target the bacterial chromosome, and constructed the engineered phage (crPhage). In vitro experiments, crPhage can infect C. difficile within 2 h, reduce the number of colonies by 3 log orders, and its bactericidal effect is significantly better than that of the wild-type phage. In the CDI model, the mice were given crPhage orally, and the number of bacteria in the feces was reduced by 10 times two days after infection. Notably, the proportion of surviving lysogens in the crPhage group was 10%, significantly lower than that in the wild-type phage group (81%). In addition, the histopathological scores of the cecum and colon of the mice in the crPhage group were similar to those of the uninfected control group, which proved that the CRISPR-Cas system effectively suppresses the formation of lysogens and reduces tissue damage [46].

#### 3.1.2. Helicobacter Pylori Infection

*Helicobacter pylori* is a Gram-negative bacterium that can colonize the gastric mucosa, with a microaerobic environment, and the global infection rate is as high as 50% [40]. This is the main cause of lymphoma, peptic ulcer, and chronic gastritis [47]. At present, the clinical eradication strategy is to use antibiotics, proton pump inhibitors, and other drugs to form a quadruple or triple regimen [35]. However, the incidence of antibiotic resistance is increasing, and the effectiveness of traditional treatment has declined to less than 80% in many areas. At present, the incidence of resistance to levofloxacin, metronidazole, and other drugs is more than 15% in some regions, which is the main reason for the failure of clinical treatment [41]. In this case, phage therapy has attracted the attention of researchers, which can accurately target pathogens without damaging the intestinal microflora, and has a good prospect [40].

At present, the research on phages against Helicobacter pylori is in its infancy, and the research focuses on the isolation of phages from environmental samples and the characterization of their characteristics. In the study, researchers used the double-layer agar method to isolate lytic phages from hospital sewage and other sources. Khosravi et al. isolated a lytic phage from hospital sewage, analyzed the protein profile with the help of sodium dodecyl sulphate-polyacrylamide gel electrophoresis (SDS-PAGE), and found that the characteristic proteins were 58 kDa and 64 kDa, respectively [48]. Electron microscopy was used to confirm its morphology, but it was also pointed out that phage infectivity decreased significantly in the purification process [48]. Similarly, in a study of the Sharkia Governorate of Egypt, three phages were isolated from sewage, but their infectivity decreased significantly after purification, indicating that phage stability needs improvement [49]. Although the sample size of this experiment was small, it nevertheless highlights the bottlenecks in phage therapy.

Genomic research has deepened our understanding of the role of H. pylori phages in bacterial evolution. In 1990, scholars used electron microscopes to observe clinical isolates and found phage particles at different stages of development, thereby proving that H. pylori contained temperate phages [22]. With the rapid development of sequencing technology, scholars have analyzed the genomes of H. pylori from different regions and have found that the presence of phages significantly impacts the adaptability and genetic diversity of H. pylori. For example, the KHP30 and KHP40 phages isolated from Japanese patients have been sequenced [50]. The major capsid protein of this phage assembles into exceptionally stable hexamers and pentamers. It also has “P-loop” structures to strengthen structural stability. The major capsid protein has a unique protruding loop that extends to the neighbouring subunit, stabilizing hexagonal capsomeres. And the capsid is decorated with trimeric cement protein, thereby enhancing the particle stability by connecting these capsomeres. The structures reveal the mechanism of capsid stability under highly acidic conditions [50].

Verma et al. (2025) [51] conducted a randomized controlled trial, which is the first systematic evaluation of the effectiveness and safety of phage combination therapy for Helicobacter pylori infection. The study was conducted in two tertiary hospitals in northern India, and 50 patients with dyspepsia were selected, all of whom were Helicobacter pylori-positive by the rapid urease test (RUT). The participants were randomly assigned to two groups: the SOC group (*n* = 24) and the phage group (*n* = 26). The former group used PPI, amoxicillin, and clarithromycin for 14 days, and the latter group used the same drugs as the former group and added Ganges River phage, which was given twice a day, 100 mL each time. The phage preparation was filtered through a 0.22 μm membrane and was found to contain a large number of natural phages. The eradication rate of the SOC group was 66.7%, and that of the phage group was 69.2%; there was no significant difference between the two (*p* = 1.000). However, in terms of quality of life and symptoms, the phage group looks more promising. The adverse reactions in the two groups were mild, including gastritis, belching, and nausea, and no serious adverse reactions occurred. The study further shows that oral phage therapy has good safety and tolerance [51].

Although orally administered natural bacteriophages do not significantly enhance the eradication rate of Helicobacter pylori, this disappointing clinical outcome can be explained at a fundamental biochemical level. The highly acidic environment of the stomach (pH 1.5–3.5) not only induces denaturation of the capsid proteins but also causes irreversible covalent cross-linking of the phage genome, leading to the complete inactivation of the vast majority of unprotected phages before they reach the mucosal target [43]. However, a small number of phages can survive at low pH (In 2022, a study found a phage named HPy1R that remained stable at 37 °C from pH 3 to 11 for 24 h in standard assays [52]).

The inactivating effect of potent gastric acidity upon bacteriophages suggests that three principal modalities are involved in this complex process. The denaturation of capsid proteins indicates that the spatial integrity of the phage’s protective shell is critically compromised under acidic conditions. Moreover, weak intermolecular forces and secondary bonding architectures, such as hydrogen-bonding interactions and hydrophobic-mediated aggregation, appear vulnerable to increased proton concentration. Given that hyperacidic milieus produce perturbation among these stabilizing factors, aberrancies in the tertiary and quaternary organization demonstrate measurable disruption within the proteins that constitute the protective shell enveloping nucleic acids. Capsid denaturation exposes genomic components to acidolytic processes. Furthermore, once the capsid matrix undergoes disintegration, the key vulnerabilities of genetic substrates indicate accelerated loss of infectivity [53]. This primary denaturative assault appears to represent the most evident operative axis among the three modalities identified. In light of these findings, exposure-induced compromise of nucleic acid components suggests that low-pH environments exert profound inactivating effects on phage viability.

Nucleic acid-protein cross-link formation shows a second operative axis less evident than the primary assault. What occasions irreversible functional attrition of the genome demonstrates a process in which chemical bridges form between discrete regions of chromatin and adjacent capsid elements [53]. Moreover, at intermediate pH values between 4 and 7, non-covalent associations preferentially occur between guanine-cytosine sequences of the viral chromosome and residues such as aspartate and glutamate. Therefore, when ambient hydrogen ion activity approaches or falls below the threshold corresponding to pH 4, the results demonstrate that re-neutralization precipitates the transformation of reversibly oriented linkages into fixed, covalent ones. Imine architecture irreversibly links chromatin to capsid proteins. The perpetuity conferred by such covalent bonds indicates a profound enough compromise to appear measurable as dramatic decrements in biological assays. The higher the GC-content, the more sensitive the phage is to the action of H(+)-ions. The acidic inactivation of virions involves acidifying the medium to a pH below 4 and then neutralizing it to pH 8, which leads to the formation of covalent DNA-protein cross-links of the Schiff base type. The study examined how the survival of bacteriophages with different GC contents changed after incubating in media of varying acidity, followed by neutralization. It was shown that the higher the GC content, the more sensitive the phage is to H(+)-ion action. The extremely low pH environment of gastric acid is the primary barrier limiting the efficacy of oral phage therapy. Biophysical analyses indicate that phages with higher genomic GC content are more sensitive to proton concentration; exposure to an environment with a pH below 4 followed by neutralization leads to the irreversible formation of Schiff base-type covalent cross-links between viral DNA and capsid proteins, resulting in the permanent inactivation of the viral particles [53].

Thus, increases in genomic GC ratios indicate heightened fragility, linking sequence composition directly to fate during passage through strongly acidic microenvironments such as the mammalian stomach cavity. GC content links acid susceptibility in viral genomes.

Although the RCT by Verma et al. (2025) [51] demonstrated that the combination of phages and standard therapy was well tolerated, eradication rates did not show a significant improvement. This suggests that oral administration of natural phages alone is unlikely to achieve the expected bactericidal effect in the real-world gastric acid environment [51]. This outcome is not coincidental but results from three main obstacles: inactivation by gastric acid [43,53], inadequate mucosal penetration [54,55], and a narrow host range [56]. It indicates that current methods of phage preparation and administration are still insufficient to replace or meaningfully enhance first-line eradication regimens.

Existing studies typically attribute phage inactivation to low pH; however, other mechanisms may also contribute during *H. pylori* treatment [53]. Limitations of in vitro experiments include the use of buffered systems that do not simulate dynamic pH changes and the gastric mucus barrier. This creates a significant gap between in vitro activity and actual in vivo efficacy—highlighting a major flaw in current research designs [54].

Additionally, most isolated *H. pylori* phages are temperate [22], posing potential risks of transferring virulence and resistance genes [57,58]. There is a serious shortage of clinical-grade, strictly lytic phages, a key obstacle hindering translation into clinical therapy.

In summary, *H. pylori* phage therapy has not yet reached clinical applicability. Most research focuses excessively on isolation and characterization [48], while neglecting three critical engineering modifications—gastric acid protection [59,60], targeted delivery, and host range expansion [61,62]—which results in a substantial disconnect between basic research and clinical needs.

#### 3.1.3. Other Pathogenic Bacterial Infections

Certain pathogens suggest that acute infectious diarrhea is significantly associated with bacterial infections, including *Escherichia coli*, *Shigella* and related organisms. Phages demonstrate rapid bactericidal activity against these key pathogenic bacteria. Furthermore, T156 phage demonstrates significant bactericidal activity under the relevant experimental conditions [63]. In light of the evidence, the significant phage cocktail suggests that combined formulations yield comparable bactericidal results [64]. Antibiotic-phage combinations show synergistic effects and eliminate biofilms [65].

Clinical trials suggest an acceptable safety profile, given evidence from 1963 in the former Soviet Union involving 30,769 children, indicating that phage administration helps prevent Shigella-associated diarrhea [66]. The significant safety trial findings indicate that 15 healthy individuals who orally administered T4 phage showed no adverse reactions and that the phage was excreted in feces in a dose-dependent manner [67]. Furthermore, in the first phase of the trial, 10 people took the drug orally for 7 consecutive days, with only 50% reporting mild gastrointestinal symptoms and no serious adverse reactions [67]. In light of these findings, the study of the safety and tolerance of oral phage therapy suggests no significant change in the composition of the intestinal microbiota. Evidence shows no improvement found. However, the significant randomized controlled trial conducted in Bangladesh suggests that phage therapy showed no meaningful effectiveness in children with *E. coli* diarrhea, and the key results indicate no significant improvement in clinical practice. 120 children were selected and randomly divided into three groups: one group given a cocktail of T4-like coliphage, one group given a Russian coliphage preparation, and the other group given a placebo. Notwithstanding these results, the four groups were treated for four consecutive days without adverse reactions, and there was no significant difference between the phage treatment group and the standard care group in diarrhea frequency, stool output, or other parameters. Given that the microbiological findings show that only 60% of children were confirmed to be infected with E. coli, *E. coli* accounted for less than 5% of the total number of bacteria in the feces, and the titer increased only transiently and not significantly. The findings of this study underscore the need for further research to understand the complex interactions between phages, the gut microbiota, and the host immune system in the context of diarrheal disease. The complex interplay between phage preparation, the target pathogen, and the gut environment likely determines the efficacy of oral phage therapy. A deeper understanding of these interactions is critical for the rational design of future phage therapy trials. Future studies should focus on optimizing phage dosing, timing, and delivery, as well as elucidating the pharmacokinetics and pharmacodynamics of phages in the complex gut ecosystem. Their results highlight that simply administering phages orally is not sufficient to achieve therapeutic success in all contexts, and that a more nuanced understanding of phage ecology within the gut is required. [68].

#### 3.1.4. Critical Analysis

Current studies have demonstrated the therapeutic potential of phage therapy against gastrointestinal infections such as *C. difficile* and *H. pylori*, providing proof-of-concept for precision, microbiota-sparing antibacterial intervention. However, the field remains limited by an overreliance on idealized in vitro models that fail to simulate extreme gastric environments, the high prevalence and genetic risks of temperate phages, and the inherent inability of natural phages to overcome physical barriers like spores and mucus. Most studies focus on passive phage isolation and characterization rather than biochemical barrier overcoming, host-spectrum expansion, and synthetic engineering optimization. To achieve clinical translation, three key issues must be addressed: smart delivery systems for gastric acid protection, AI or CRISPR-assisted modification to overcome narrow host ranges, and a paradigm shift toward synthetically engineered strict lytic phages.

### 3.2. Inflammatory Bowel Diseases (IBD)

#### 3.2.1. Inflammatory Bowel Disease Pathogenesis

Inflammatory bowel disease (IBD), primarily encompassing Crohn’s disease (CD) and ulcerative colitis (UC), suggests an exceptionally complex pathogenesis. The etiology of IBD is complex and multifactorial, involving environmental, microbiota, genetic, and immunological factors that alter the organism’s molecular basis. Understanding the imbalanced gut microbiota in disease conditions is essential for uncovering disease mechanisms and developing effective therapies [69,70].

Genetic factors are crucial in determining susceptibility to IBD. They lay the groundwork for understanding abnormal immune responses to gut microbiota. Polygenic risk is a major factor. Genome-wide association studies (GWAS) have identified over 200 loci associated with IBD susceptibility. These genes are mainly involved in immune regulation (e.g., IL23R), autophagy (e.g., ATG16L1), and bacterial recognition (e.g., NOD2) [71,72]. Despite these genetic findings, environmental and microbial factors also significantly contribute to overall IBD susceptibility.

Research shows intestinal immune dysregulation drives inflammation.

Innate immune hyperactivation is a crucial mechanism, as Toll-like receptors (e.g., TLR2, TLR4) are significantly upregulated in the intestinal mucosa of IBD patients. This upregulation leads to excessive recognition of luminal bacteria, activating the NF-κB signalling pathway and resulting in the release of numerous pro-inflammatory cytokines (e.g., TNF-α) [65]. Since adaptive immune imbalance is a major factor, the findings imply that dysregulation of the CD4^+^ T cell subsets is a primary cause of intestinal injury. Crohn’s disease (CD) is mainly driven by a Th1/Th17-type response, whereas ulcerative colitis (UC) shows an atypical Th2-type response [71]. Moreover, Treg function is impaired, and inflammation suppression is compromised [71].

The presence of oxidative stress indicates an imbalance in which excessive production of ROS by intestinal epithelial and immune cells overwhelms antioxidant capacity, directly damaging the intestinal mucosa and further amplifying significant inflammatory signals [71]. Moreover, the intestinal epithelium serves as a key physical barrier separating the massive load of luminal microorganisms from the internal milieu, and IBD patients exhibit aberrant expression of tight junction proteins in intestinal epithelial cells [5,7]. Furthermore, this increases paracellular permeability, commonly referred to as “leaky gut.” These findings imply that heightened apoptosis or necroptosis of epithelial cells weakens the epithelial layer’s integrity. Importantly, these disruptions allow bacteria and their products to cross the barrier, triggering activation of submucosal immune response cells [71]. Disruption of the gut microecosystem shows that IBD pathogenesis is centrally affected. The species richness and diversity of the gut microbiota in IBD patients appear substantially lower than those in healthy individuals. Furthermore, these shifts involves a reduction in beneficial bacteria and an increase in potentially harmful ones. The results show a significant decline in bacteria like *Faecalibacterium* spp. and *Roseburia* spp., which produce anti-inflammatory SCFAs. This implies that an increase in bacteria with pro-inflammatory traits, such as adherent-invasive Escherichia coli (AIEC), occurs [66,67]. AIEC proliferation is associated with IBD-related dysbiosis outcomes [73,74].

#### 3.2.2. The Pathogenic Mechanism and Pathophysiological Role of Bacteriophages in Inflammatory Bowel Disease (IBD)

Just as mentioned earlier, in a healthy body, phages can reduce reactive oxygen species (ROS) levels [35], block T cell activation, and inhibit the NF-κB signalling pathway, thereby playing an anti-inflammatory role [36]. However, the significant findings from recent multi-omics evidence suggest that this view requires revision. Dysbiosis of endogenous phage communities is strongly associated with Inflammatory Bowel Disease (IBD). However, the current literature primarily indicates a correlative link between changes in the phageome and the disease state, rather than establishing phages as direct pathological drivers that induce intestinal inflammation and tissue damage [30,75,76]. A strong link between dysbiosis of endogenous phage communities and the disease state of IBD, suggesting they may act as potential modulators.

In the gut microbiome of IBD patients, a prominent virological feature is the abnormal expansion of the class *Caudoviricetes*. Given that the evidence demonstrates this expansion, it strongly correlates with a sharp decline in bacterial diversity. This expansion demonstrates an important association with exacerbated intestinal inflammation. Intact phage particles interact with the intestinal mucosal surface upon impairment of the epithelial barrier, exposing them directly to innate immune cells in the lamina propria [77].

Bacteriophages can alter mucosal immunity, thereby impacting mammalian health. Once phages are inside endosomal compartments, *Lactobacillus* spp., *Escherichia* spp., and *Bacteroides* spp. bacteriophages and phage DNA stimulate IFN-γ via the nucleotide-sensing receptor TLR9. The resultant immune responses were both phage and bacteria-specific [78]. Bacteriophages act as active immunomodulators in the gut. Upon internalization, phage DNA can trigger innate immune responses by activating the TLR9-MyD88 signalling axis in mucosal immune cells. This pathway promotes the nuclear translocation of NF-κB and drives the excessive release of pro-inflammatory cytokines, particularly IL-6 and IFN-γ, which collectively exacerbate intestinal inflammation. Together, these observations position phage DNA as an unexpected trigger of innate immune activation in the IBD-susceptible host. Indeed, increasing bacteriophage levels exacerbated colitis via TLR9 and IFN-γ signalling, and mucosal IFN-γ levels positively correlate with bacteriophage levels, suggesting that phage-derived nucleic acids contribute directly to the aberrant immune responses characteristic of IBD [79].

Experimental models have begun to dissect the causal relationships underlying these observations. In preclinical mouse studies, treating germ-free mice with bacteriophages leads to immune cell expansion in the gut [78]. Reducing the burden of viral DNA within the intestinal lumen attenuates TLR9-dependent signalling, suggesting that the presence of phage genetic material is necessary for sustained immune activation. Conversely, exogenous administration of CpG-containing oligodeoxynucleotides recapitulates the inflammatory phenotype, whereas treatment with TLR9-specific antagonists—such as ODN 2088—may attenuate phage-driven pathology. Collectively, phage DNA plays a central, non-redundant role in triggering innate immune dysfunction in the context of IBD [79].

The immunological consequences of phage internalization, however, extend beyond the TLR9–MyD88 axis. Amplification of downstream cytokine responses is evident through the marked activation of T helper 1 (Th1) lymphocytes and natural killer (NK) cells. These populations secrete copious amounts of interferon-γ (IFN-γ), a cytokine increasingly recognized as a critical mediator of mucosal damage and disease perpetuation in Crohn’s disease pathogenesis [80]. As detailed in a recent comprehensive review, IFN-γ exerts pleiotropic effects on both innate and adaptive immune compartments, orchestrating a pathogenic immune milieu that disrupts intestinal epithelial integrity and sustains chronic inflammation [80].

At the epithelial surface, IFN-γ acts directly on intestinal epithelial cells to downregulate transcription and expression of key tight junction proteins, including claudins and occludins. The resulting widening of intercellular spaces increases paracellular permeability, facilitating the translocation of luminal antigens, bacteria, and additional phage particles across the compromised barrier. This influx, in turn, perpetuates and amplifies immune activation within the mucosal compartment. Notably, “small-molecule Janus kinase (JAK) inhibitors targeting various nodes of the IFN-γ axis” have been explored for their capacity to “attenuate IFN-γ-driven inflammatory cascades”, reinforcing the role of JAK–STAT signalling in promoting proinflammatory gene transcription and epithelial injury [80].

IFN-γ destabilizes vascular endothelial cadherin (VE-cadherin) and it dismantles adherens junctions. Thus, massive numbers of leukocytes undergo transendothelial migration into the inflamed tissue. Moreover, the IBD pathological microenvironment appears particularly susceptible to external disturbances, such as infections or antibiotic exposure. Given that evidence demonstrates this originally protective immune response becomes reprogrammed, the shift toward a pathological, hyperactive Th17 cell response ultimately leads to excessive interleukin-17 (IL-17) production and severe mucosal damage [76]. IBD links to lysogenic conversion, horizontal transfer of virulence factors [81], and disruption of Bacteriophage Adherence to Mucus (BAM) [82,83,84].

**Figure 2:**
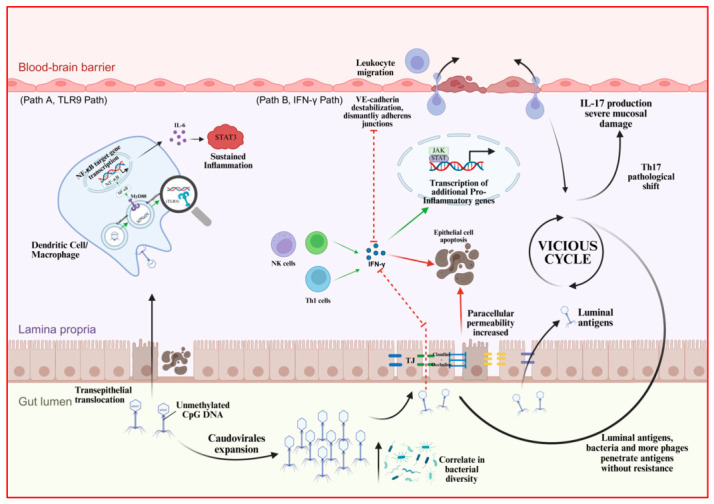
Mechanisms of phage-induced pathology in inflammatory bowel disease (IBD). This schematic outlines how dysbiosis of the endogenous virome exacerbates intestinal inflammation. Path A illustrates the TLR9-mediated pathway where internalized phage DNA activates innate immune cells, triggering pro-inflammatory cytokine release. Path B details the subsequent IFN-γ signalling cascade, which dismantles epithelial adherens junctions (e.g., VE-cadherin downregulation) and increases paracellular permeability. This “leaky gut” effect creates a vicious cycle, allowing massive luminal antigens and additional phages to translocate into the lamina propria, further driving a pathological Th17 immune shift. Created in BioRender. Shaokun Zhang. (2026) https://BioRender.com/hcw0lp5 (accessed on 15 March 2026).

The two-way shows that phages change IBD, as shown in Figure 2.

#### 3.2.3. Preclinical Basic Research on Phage Therapy in Inflammatory Bowel Disease (IBD)

The endogenous temperate phages that proliferate in IBD patients are closely correlated with disease progression, whereas the exogenous lytic phages we use for clinical treatment are carefully screened or engineered. The core principle of the treatment is to employ beneficial exogenous phages to precisely eliminate pathogenic bacteria, thereby breaking the original vicious cycle of inflammation.

Endogenous virome dysbiosis suggests that significant pathogenic disruption underlies gut dysfunction [85]. Broad-spectrum antibiotics can inflict severe collateral damage on the delicate gut microbiota [86,87]. This includes secondary complications such as *Clostridioides difficile* infection [88]. Exogenous strictly lytic phages represent a precision microbiome intervention [89,90]. Preclinical studies suggest that phages are effective in treating IBD. Various acute and chronic colitis models provide a robust theoretical and experimental foundation for evaluating these approaches [23,91]. The DSS-induced colitis model effectively replicates epithelial damage and immune hyperactivation seen in human ulcerative colitis (UC). Its reproducibility supports its status as the gold standard for testing new IBD treatments. Studies confirm the effectiveness of oral phage cocktails, and consistent results across experiments indicate their reliable anti-inflammatory effects and potential to protect the intestinal barrier. Despite some methodological differences, mice can ingest DSS through drinking water, thereby damaging the colonic epithelium, and gavage delivery is effective [92]. Purified phage mixtures can be administered orally via gavage after epithelial disruption. Histopathological analysis with H&E staining indicates that phage therapy significantly decreases the Disease Activity Index (DAI). Phage therapy has been observed to attenuate colon shortening and help improve mucosal ulceration. Since histological data support barrier restoration, phage treatment reduces crypt destruction and preserves goblet cell loss [92]. Research shows molecular repair occurs. Western blot and qPCR analyses reveal that phage intervention inhibits the production of proinflammatory cytokines, including TNF-α, IL-6, and IL-1β. Based on these molecular findings, phage therapy increases the expression of essential tight junction proteins, particularly Zonula Occludens-1 (ZO-1) and claudin-1 [93]. This process physically repairs the damaged intestinal barrier. Flow cytometry confirms certain phages, such as FPSP6, have immunomodulatory capabilities. These phages can bind to PRRs on antigen-presenting cells (APCs). Furthermore, the results imply that these phages interact with pattern recognition receptors (PRRs) on APCs to modulate innate immunity. Consequently, the evidence points to this mechanism potentially helping to eliminate targeted pathogens such as non-typhoidal Salmonella [94]. In light of these key findings, phage intervention appears to attenuate pro-inflammatory cytokine cascades while maintaining a favourable safety profile with no evident systemic toxicity in vivo [94].

Adherent-invasive Escherichia coli (AIEC) indicates that mucosal colonization plays an important role in the development of Crohn’s disease (CD), as findings show significant enrichment in the ileal mucosa of affected patients [95,96]. AIEC binds to epithelial cells through CEACAM6 receptors, indicating a mechanism for mucosal invasion. AIEC can survive and replicate inside macrophages, potentially contributing to chronic granulomatous inflammation. Since these pathogenic processes are supported by the evidence, effectively clearing AIEC remains a critical focus in IBD phage research. Studies with gnotobiotic and transgenic mice colonized with the AIEC strain LF82 demonstrate that phage cocktails significantly reduce bacterial load magnitude [97]. This treatment effectively reduces DSS-induced intestinal inflammation, indicating that phage-based approaches are promising. Phages with unique mechanisms have been identified, implying that direct lysis is only one aspect of their therapeutic action. For example, phage HER259 targets AIEC strain NRG857c, affecting more than just lysis—potentially modulating virulence. Evidence shows AIEC can adapt genomically under selective pressure. The *fimS* promoter also undergoes a sequence-specific inversion to an off state, likely blocking FimH adhesin expression, which is crucial for epithelial attachment. Without adhesion, AIEC cannot invade the mucosa, possibly leading to a complete loss of pathogenicity. Removing HER259 causes the *fimS* promoter to revert, increasing the likelihood of colitis recurrence. Continuous phage pressure appears to maintain remission, and in vivo trials indicate that HER259 works synergistically with low doses of budesonide, potentially enhancing therapeutic efficacy [98]. Based on these results, this approach also helps lower the significant systemic side effects of corticosteroids, which are an important factor to consider for clinical use.

Because of multidrug-resistant bacteria, IBD complications are increasingly difficult to address through conventional approaches. Biological lysis alone is not enough for complex clinical challenges. Since carbapenem-resistant *Klebsiella pneumoniae* is a high-priority threat listed by the WHO, gut colonization leads to deadly inflammatory responses. Consequently, these significant findings have led scientists to develop an engineered phage complex, P510@CeO2, to address these urgent issues. CP links phages to nanoparticles. This precision approach covalently attaches the CRKP(Carbapenem-Resistant *Klebsiella pneumoniae*)-specific phage P510 to catalytic cerium oxide nanoparticles in a targeted way. The research team likely confirmed the complex’s nanostructure using transmission electron microscopy. The data show that hydrodynamic size and zeta potential were measured via UV-vis spectroscopy and dynamic light scattering. The complex remains highly stable in simulated gastric and intestinal fluids. CP maintains stability in these fluids. And therapeutic efficacy was rigorously tested in IBD mice infected with hypervirulent CRKP. In vitro soft agar assays and 3D confocal laser microscopy confirmed that CP effectively eradicates CR-hvKP21. The complex can also quickly destroy resilient bacterial biofilms. Furthermore, the transcriptomic and gene expression analyses suggest that CP downregulates capsule-encoding genes. CP also suppresses MDR pumps. This suppression reduces the emergence of phage-resistant mutants at the genomic level [14].

#### 3.2.4. Clinical Research of Phage Therapy in IBD

Theoretical efficacy supports further investigation. Clinical translation requires rigorous validation of human pharmacokinetics (PK), pharmacodynamics (PD), and safety assessments [99]. As mentioned earlier, the high-acid environment of the stomach has made it a major challenge to pass through the stomach and reach the intestines. BiomX has developed experimental phage cocktail therapies BX002-A and BX003 to address this issue. Both BX002-A and BX003 target specific strains of Klebsiella pneumoniae. This bacterium is abundant in the gut microbiome of patients with IBD and PSC, where it can damage the intestinal epithelial barrier, leading to ‘Leaky Gut’ and severe immune-mediated inflammatory responses. BX002-A is an early oral candidate drug for this therapy and was the first to enter human clinical trials. BX003 is an evolved version of it. In November 2020, BiomX merged its R&D pipelines for IBD and PSC to develop broader-spectrum, formulation-optimized follow-up products, collectively named BX003. This experiment was a randomized, single-blind, placebo-controlled trial involving healthy adult volunteers; participants received twice-daily doses of 2.8 × 10^10^ PFU of an ultra-high-titer phage cocktail while also taking a proton pump inhibitor (esomeprazole) to neutralize stomach acid. Under the protection of the proton pump inhibitor, orally administered phages survive and reach the lower digestive tract, with high levels of active phages detected in stool samples, without causing systemic inflammation or neutralizing antibody responses. This study was the first in the medical field to demonstrate the effectiveness of ‘oral live phages’ and marked the beginning of a new era in ‘precise editing of the gut microbiome.’ It offers new hope for patients who have long relied on immunosuppressants with severe side effects—not just suppressing the immune system but directly eliminating pathogenic bacteria at their source [100].

In addition to phage therapy designed by BiomX, Intralytx also makes other methods of phage therapy in the clinical experimental stage, as shown in Table 3.

#### 3.2.5. Current Limitations and Challenges of Phage Therapy in IBD

Phage therapy, including both natural and engineered types, shows great promise in tackling multidrug-resistant infections and restoring gut health. However, moving from lab research to everyday clinical practice still faces many obstacles [100]. These include fundamental biological, immunological, regulatory, and manufacturing challenges. Evidence of rapid evolutionary pressure suggests that therapeutic phages impose a strong selective force on target bacteria, similar to how antibiotic overuse promotes the development of superbugs [97]. Bacteria can develop resistance quickly in three ways. Firstly, Horizontal Gene Transfer (HGT) is the primary mechanism for the rapid and widespread acquisition of resistance. HGT allows bacteria to share genetic material (including Antimicrobial Resistance Genes, or ARGs) not just with their offspring, but with neighbouring bacteria—often across entirely different species [101]. Secondly, the use (and misuse) of antibiotics creates a massive evolutionary bottleneck. When an antibiotic is introduced into an environment (like the human gut), it quickly eradicates the susceptible bacterial populations. This clears the ecological niche, completely removing competition for resources. The few bacteria that possess resistance genes are left to rapidly multiply and colonize the newly available space, quickly shifting the entire population from susceptible to resistant [102]. Thirdly, bacteria often aggregate into biofilms—dense communities encased in a self-produced protective polymeric matrix. Within a biofilm, bacteria are physically shielded from antibiotics and immune cells. Furthermore, bacteria deep within the biofilm often enter a dormant metabolic state. Since many antibiotics only target actively dividing cells, these dormant bacteria survive the treatment and can quickly repopulate the environment once the antibiotic is removed [103].

Additionally, bacteria utilize multiple defences to withstand this evolutionary arms race. A key strategy involves genetic mutations that alter or diminish cell surface receptors. Bacteria also use restriction-modification systems to identify and cleave foreign phage DNA upon injection. Consequently, these combined resistance mechanisms complicate efforts to attain consistent therapeutic success throughout treatment cycles. Phage resistance continues to evolve quickly [97].

In addition, the CRISPR-Cas adaptive immune system is even more complex, as bacteria incorporate phage DNA fragments into CRISPR arrays to create a molecular memory. Despite these findings, Cas nucleases have the ability to accurately identify and neutralize matching phages upon re-infection, implying that single phages fail within days. Therefore, clinicians need to use updated cocktails or remodel receptor-binding proteins through directed evolution to stay therapeutically relevant [96,98]. In light of these findings, such demands mean that bedside treatment logistics are significantly complicated by these evolutionary challenges.

Furthermore, the core advantage of phage therapy—extreme strain-level specificity—also demonstrates its greatest weakness for broad deployment. Unlike broad-spectrum antibiotics, phages typically infect only a few specific strains within a species, which suggests that IBD patients present considerable diagnostic challenges [97,100]. Pathogenic *E. coli* strains differ significantly in genotype and phenotype between patients, such that a cocktail effective for one patient fails for another [97]. Given that the evidence demonstrates these diagnostic demands, clinicians must isolate the specific pathogen and perform time-consuming phagogram sensitivity tests [100]. Personalized diagnosis required.

However, the human immune system treats exogenous phages as foreign antigens, triggering strong humoral immune responses upon high-titer delivery. The host produces neutralizing antibodies, such as secretory IgA and serum IgG, against phage capsids or tail fibres. These antibodies cause steric hindrance and accelerate metabolic clearance via the reticuloendothelial system. Therefore, a reduced in vivo half-life diminishes the efficacy of repeated dosing across treatment cycles. Immunity limits repeated dosing [104].

Another safety concern involves the rapid, synchronized lysis of Gram-negative pathogens, which suggests that significant amounts of bacterial endotoxins (LPS) and exotoxins accumulate dangerously. In IBD patients with compromised intestinal barriers, these toxins provide a clear pathway into the circulation [100,105]. Furthermore, this process appears to trigger Systemic Inflammatory Response Syndrome (SIRS) or septic shock [105]. In light of the results, these outcomes pose a direct and critical threat to life. Lysis releases toxins. Toxins enter circulation. SIRS or septic shock follows.

Not all phages are suitable for therapy, and temperate (lysogenic) phages, while abundant in nature, demonstrate poor therapeutic candidacy [57]. Without strict screening, temperate phages appear to integrate their DNA into the host genome rather than lyse the bacteria. In inflamed gut biofilms, dangerous transduction activity [58]. Given that the data demonstrates this pathway acts as a highway for the horizontal transfer of antibiotic resistance (AMR) genes and virulence factors, candidates require deep whole-genome sequencing and rigorous bioinformatic vetting. Screening shows the absence of integrases, toxin genes, or resistance determinants. Therefore, this screening process entails high technical and temporal costs that appear unavoidable [57,58].

#### 3.2.6. Critical Analysis

Existing research has demonstrated the therapeutic potential of phage therapy in treating inflammatory bowel disease (IBD)—particularly against pathogenic strains such as adherent invasive Escherichia coli (AIEC) and in modulating the host immune response—providing proof of concept for precision antimicrobial interventions. However, the field remains constrained by the blurred distinction between correlation and causation in viral dysbiosis, significant differences between acute animal models and chronic human pathology, and the rapid emergence of bacterial resistance. Most studies have focused on observational characterization and early safety validation rather than overcoming the complex physical mucosal barrier, engineering optimizations to circumvent bacterial defence mechanisms, and investigating the dynamic properties of formulations. To achieve clinical translation, key challenges must be addressed: shifting toward the active engineering of phages with broader host ranges; developing advanced targeted delivery systems to penetrate the intestinal mucus barrier; and establishing an adaptive regulatory framework with rigorous quality control to manage personalized live biotherapeutics.

### 3.3. Cirrhosis-Associated Gastrointestinal Infections

#### 3.3.1. Pathophysiological and Clinical Phenotype Differences: Cirrhosis-Related vs. General Gastrointestinal Infections

Gastrointestinal (GI) infections represent conditions that suggest a highly significant burden across clinical settings [106]. Cirrhosis—an end-stage chronic liver disease—significantly alters the way these infections progress [107,108]. This transformation influences their etiology, microbial composition, intestinal barrier integrity, and systemic immune response [108]. Since these distinctions seem important for clinical practice, understanding the differences between cirrhosis-related GI infections and those in immunocompetent individuals continues to be essential [109,110,111]. Cirrhosis exhibits specific infection patterns. Understanding these patterns is essential for creating targeted microbiome treatments, like phage therapy [112]. Understanding these differences also aids in developing more targeted therapies [113]. Based on the data, the findings highlight that variability in infection sources, patterns, and pathogen characteristics is crucial for further research. Consequently, understanding this heterogeneity seems important for informing clinical decisions. Infections vary depending on the host context [111].

In immunocompetent individuals without structural abnormalities, GI infections typically result from sudden invasion by external pathogens. These infections are usually self-limiting and limited to the intestinal lining. Key clinical signs include sudden fever, abdominal cramps, watery or bloody diarrhea, and dehydration. However, it appears that cirrhosis-related GI infections are primarily caused by endogenous factors rather than external sources [107]. Cirrhosis drives endogenous infection. The difference between cirrhosis-related and general gastrointestinal infections is shown in Table 4.

As fibrous scar tissue replaces the liver parenchyma, patients experience severe gut dysbiosis and small intestinal bacterial overgrowth (SIBO) [107,115]; a pattern of progressive microbial imbalance over time. Since the results support this trend, the key features of this progression include a significant reduction in beneficial bacteria like *Lachnospiraceae and Ruminococcaceae (phylum Firmicutes)* [115]. Furthermore, the overgrowth of Gram-negative bacteria, such as *Enterobacteriaceae (phylum Proteobacteria)*, and certain Gram-positive bacteria like *Enterococcus* and *Streptococcus*, appears to have a negative effect on the organism during their expansion [115]. When commensals decline, pathogens may expand. However, the key findings imply that the pathogens involved originate from existing colonisers, known as pathobionts, in the host’s gut rather than from external invaders [107,116]. Although interactions among many pathobionts may have a negative influence, Escherichia coli, Klebsiella pneumoniae, and Enterococcus faecalis play key roles [115]. Decompensated cirrhosis is associated with fungal overgrowth, particularly of *Candida* spp., especially in patients on extensive prophylactic antibiotics. This cross-kingdom imbalance further increases the already high risk of infections [117]. Fungal overgrowth can therefore worsen the risk of infection. Moreover, gastrointestinal (GI) infections and healthy epithelial and vascular barriers can effectively confine pathogens and toxins within the intestinal lumen [118].

Barriers restrict systemic spread. Although pathogens can cause localised inflammation and temporary changes in permeability, the damage is typically superficial and reversible. The gut-associated lymphoid tissue (GALT) quickly clears invading pathogens, thereby preventing systemic spread. Moreover, this clearance mechanism effectively contains localised pathogenic activity. GALT’s rapid pathogen clearance prevents spread [119].

Systemic structural and metabolic issues play a role in chronic leaky gut syndrome through various underlying mechanisms. Portal hypertension causes splanchnic vasodilation, venous congestion, and bowel wall edema, which weaken mucosal mechanical integrity [107,114]. Additionally, the liver’s reduced capacity to produce and release primary bile acids highlights a critical disruption in metabolic regulation, while dysbiosis further hampers the conversion into secondary bile acids [116]. In light of the significant findings, weakened Farnesoid X Receptor (FXR) signalling suggests that reduced antimicrobial peptide expression and disruption of the apical junctional complex, including tight junction proteins such as Zonulin [120], appear to follow. FXR signalling links to barrier collapse. Furthermore, the loss of commensal bacteria indicates that short-chain fatty acid (SCFA) production, such as butyrate, is markedly reduced, depriving epithelial cells of key energy sources [115,121]. Thus, the collapse of these barriers contributes to a profound and progressive increase in intestinal permeability [107,114]. However, the literature indicates that this barrier dysfunction is not strictly permanent and may be partially reversible if the underlying hepatic inflammation is controlled or through targeted microbiome-modulating therapies. Current evidence does not support irreversible progression, and intestinal permeability can be partially restored by targeted interventions. Following this barrier compromise, bacteria and pathogen-associated molecular patterns (PAMPs), like lipopolysaccharides (LPS), breach the intestinal wall. This suggests that harmful Bacterial Translocation (BT) likely occurs through the portal vein liver [107,108,114]. Permeability collapse causes BT.

Since immune responses in these two conditions follow different paths, immunocompetent persons show acute, self-limiting inflammatory reactions to GI infections. Pattern recognition receptors (PRRs) detect external PAMPs, triggering a cytokine cascade that recruits macrophages and neutrophils to effectively eliminate pathogens. Additionally, after the infection is resolved, inflammation decreases, and tissue repair begins [122,123,124,125].

However, in cirrhosis, the immune response is heavily distorted, a condition known as Cirrhosis-Associated Immune Dysfunction (CAID) [108]. Chronic leaky gut leads to a continuous influx of PAMPs into the liver, initially causing Kupffer cells and sinusoidal endothelial cells to develop endotoxin tolerance. This immune distortion characterizes CAID. Consequently, the evidence points to a breakdown of this tolerance during the decompensated stage, in which sinusoidal cells shift to a highly pro-inflammatory phenotype [108,126].

Under CAID, the phagocytic activity of the reticuloendothelial system (RES) suggests that a catastrophic decline occurs across immune regulatory pathways. Translocated bacteria are no longer effectively inactivated by compromised immune responses. In light of the findings, overactivated immune cells suggest a massive release of pro-inflammatory cytokines—including TNF-α, IL-1β, IL-6, and IL-18—consistent with sepsis-like systemic inflammatory response syndrome (SIRS). Given that the evidence demonstrates this chronic state exhausts immune reserves, immune paralysis or Compensatory Anti-inflammatory Response Syndrome (CARS) appears likely [106]. CARS leaves patients vulnerable to secondary infections. This compensatory state demonstrates sustained suppression of critical immune functions. Furthermore, immune reserve depletion appears to follow a progressive trajectory among affected patients [126]. Notwithstanding the CARS and immune paralysis suggest overlapping mechanistic pathways, the mortality risk appears substantially elevated in vulnerable populations. Mortality risk increases significantly.

Due to the unique nature of intestinal infections in the context of cirrhosis, bacteriophages—as viruses that target bacteria—have either beneficial or detrimental effects on the regulation of the intestinal microenvironment in such cases.

#### 3.3.2. Pathogenesis and Ecological Driving Roles of Bacteriophages in Cirrhosis-Related GI Infections

Traditional frameworks of infectious pathology suggest that the phageome, which dominates the human gut virome, offers a significant new perspective on cirrhosis-related dysbiosis [85]. Key insights from metagenomics and high-throughput sequencing suggest a move away from frameworks that focus only on bacteria. Bacteriophages—viruses that infect and lyse bacteria—serve as important natural predators of bacterial populations [127]. Given that phages participate in genetic recombination and modulate host immunity, abnormal phage activity is associated with the development of cirrhosis and related conditions like alcoholic hepatitis [127] and non-alcoholic fatty liver disease (NAFLD) [128]. Interactions between phages and bacteria influence pathogen virulence and can speed up disease progression [127].

In healthy individuals, the gut phageome maintains high diversity and a dynamic balance. This equilibrium plays an important regulatory role in controlling bacterial populations to sustain gut eubiosis [129]. Evidence from studies on fecal viromes in patients with decompensated cirrhosis, Alcohol Use Disorder (AUD), and advanced NAFLD indicates a consistent trend: a severe decline in the overall Alpha diversity of the gut phageome. Despite some specific phages increasing alongside their host bacteria, this ecological balance is disrupted in cirrhosis. Therefore, IBD is linked to decreased virome diversity, loss of core protective phages, and a selective increase in bacteriophages and eukaryotic viruses [85].

Variations in the phage community among individuals were linked to gender, country of birth, diabetes, and liver steatosis. Liver steatosis was associated with a decrease in *Lactococcus* phages r1t and BK5-T, alongside an increase in globally common Crassvirales phages, including members of the genus clusters IX *(Burzaovirus coli, Burzaovirus faecalis) and VI (Kahnovirus oralis)*. These Lactococcus phages showed strong correlations and co-occurrence with Lactococcus lactis [128]. Given that phages targeting potential pathobionts are abnormally enriched, the levels of phages attacking Escherichia coli, Enterobacteriaceae, Staphylococcus, and Streptococcus are abnormally elevated in cirrhosis and alcoholic hepatitis, with evidence indicating links to disease severity, fibrosis stages, and mortality rates [127].

In cirrhosis, environmental stressors are hypothesized to shift healthy phage–host dynamics from a lytic “kill-the-winner” model [127,128] to a “piggyback-the-winner” lysogenic state [129]. However, it is crucial to emphasize that the application of these ecological models to cirrhosis remains highly speculative and currently lacks clinical validation. While theoretical models associate this shift with the elimination of commensals and pathogen overgrowth, there is currently no direct experimental data in cirrhosis models to support the scenario that phage induction actively drives this selective dysbiosis in vivo [130,131]. Claims that stress-induced prophage reactivation leads to massive bacterial lysis and the release of abundant PAMPs (such as endotoxins) are biologically possible, but they are not firmly supported by direct in vivo evidence. While theoretical models suggest these processes could breach the compromised intestinal barrier, hyperactivate liver macrophages, and exacerbate hepatic fibrosis, these statements primarily represent mechanistic hypotheses [130,132]. Concurrently, it is suggested that translocated free phages might act as direct immunogens; host cytosolic sensors (e.g., cGAS-STING) recognizing their nucleic acids and initiating robust pro-inflammatory cascades [13,133]. Furthermore, although the synergy between indirect PAMP-mediated inflammation and direct phage immunogenicity is hypothesized to be linked with Systemic Inflammatory Response Syndrome (SIRS), robust experimental models and longitudinal clinical studies are urgently needed. Current data primarily establish a correlative link rather than a definitive causal relationship regarding phages actively driving systemic inflammation [13,84].

#### 3.3.3. Progress in Basic and Clinical Research of Phage Therapy

Traditional broad-spectrum antibiotics have significant limitations in treating cirrhosis-related infections. The rise of multidrug-resistant (MDR) bacteria poses a key concern: irreversible damage to the microbiome. Given that this therapeutic gap appears substantial, the medical community shows renewed interest in Bacteriophage Therapy [105]. Research shows phages enable precision microbiome editing. Moreover, metagenomic sequencing is a reliable method for directly pinpointing core pathogens from fecal samples [127]. Therefore, this approach appears to bypass the need for lengthy in vitro cultivation. In light of these results, key targets indicate virulent strains of E. coli, K. pneumoniae, or E. faecalis as the most relevant candidates [134]. Strictly lytic phages demonstrate effective isolation from environmental samples or phage libraries using plaque assays [105]. Evidence shows single phages face resistance. High-throughput platforms are capable of generating reliable Phagograms for research purposes. Thus, selecting multiple phages with non-overlapping resistance mechanisms demonstrates synergistic bactericidal effects in formulated Phage Cocktails [133]. Given that germ-free mice indicate a valuable model, fecal microbiota transplantation (FMT) appears essential for creating humanized mouse models [127]. Administration routes indicate oral gavage for GI infections and intravenous injection for systemic bacteremia as important considerations [105]. Data show that phages lose activity in gastric or bile acids. However, the findings suggest that advanced nanotechnology, such as liposome encapsulation, offers a reliable solution that appears to ensure phage survival through the digestive tract [59]. Furthermore, recent animal studies indicate high efficacy of phage therapy in liver disease as a key result. In light of these significant findings, Duan et al. suggest that their results provide groundbreaking evidence in alcoholic hepatitis research [127].

High mortality in these patients indicates a link to the expansion of cytolysin-secreting E. faecalis. Specific phages reduce the abundance of cytolysin-positive bacteria in humanized, ethanol-fed mice. Oral administration blocks cytolysin transport to the liver. In light of these findings, treatment appears to reduce liver steatosis, inflammation, and serum ALT/AST levels [127]. The bactericidal effect shows extreme precision. The overall Alpha diversity of the gut microbiota appears to remain intact. Antibiotics cannot demonstrate that this level of precision is achievable. Given that the results show that Gan et al. identified similar outcomes, high-alcohol-producing *K. pneumoniae* appears to drive fatty liver in Non-Alcoholic Fatty Liver Disease (NAFLD). In their preclinical murine models, targeted phage therapy was shown to reduce the abundance of this specific bacterial strain and ameliorate the associated hepatic inflammation [135].

#### 3.3.4. Clinical Transformation and Compassionate Use

Clinical translation is now actively advancing from laboratory settings toward registered trials and emergency applications [105,136]. The findings from compassionate-use cases indicate that phages have significant life-saving potential [133]. Such interventions are typically considered when top-tier antibiotics, including carbapenems, fail to clear the infection. In light of these results, phage-based approaches offer critical alternatives for otherwise untreatable patients. Phage therapy can treat infections when phages are ineffective.

A patient with a pan-resistant *A. baumannii* infection responded to treatment. After receiving FDA approval, a phage cocktail was administered via intravenous (IV) and intracystic routes, successfully clearing the infection. Notwithstanding the severity of the condition, the patient recovered without impaired liver or kidney function. Given that significant results emerged from a second case, an 88-year-old patient with multi-drug resistant (MDR) *P. aeruginosa* received targeted phage intervention after standard treatments and biliary drainage (PTCD) failed [133]. Cases show phages eliminate resistant pathogens. Thus, a Good Manufacturing Practice (GMP)-standard cocktail was injected directly through the PTCD catheter, yielding rapid improvements. Bile color normalized, and systemic inflammation markers dropped sharply within days. Furthermore, these key cases support the conclusion that phages can eliminate pathogens that remain untreatable by traditional medicine. In light of this evidence, registered clinical trials indicate that important advances are now occurring in liver and gastrointestinal (GI) diseases [127]. Data show that drug candidates targeting resistant pathogens are advancing.

Patients improved in cognitive tests related to Hepatic Encephalopathy (HE). Trials confirm that the PreforPro cocktail is highly safe [137]. Evidence from randomized controlled trials (RCTs) indicates that oral phages do not cause serious side effects. These interventions do not harm beneficial bacteria, supporting their safety. The main advancement of phage therapy lies in the precise modulation of the microbiota. Since phages target specific pathobionts with resistance genes like snipers, a healthy gut microbiome can be effectively maintained [137]. Ultimately, phages assist in restoring the microbiome to a state of eubiosis.

Furthermore, preclinical experimental models suggest that this intervention may offer benefits beyond merely clearing acute infections [127]. By potentially limiting the translocation of pathogen-associated molecular patterns (PAMPs) across compromised intestinal barriers, theoretical models hypothesize that phage therapy could help mitigate the cytokine storm linked to the gut–liver axis. However, it is crucial to note that these mechanisms are primarily observed in animal models. Assertions of clinical applications of phage therapy [109] remain highly speculative. Currently, there is insufficient clinical evidence to support these claims in human practice. Moving forward, it is more scientifically sound to view phage therapy as a promising investigational or adjunctive approach, requiring rigorous validation through large-scale, randomized clinical trials before it can be integrated into standard care protocols.

Despite existing barriers to transplant eligibility, phage therapy provides essential survival chances for end-stage patients currently ineligible for liver transplants [127]. The compelling evidence suggests that this intervention could be a pivotal moment in managing complex liver diseases. The broader clinical impact of phage-based modulation extends beyond infection control, influencing the treatment of systemic disease.

#### 3.3.5. Current Disadvantages and Limitations of Phage Therapy

Similar to IBD, efforts to standardize phage therapy for cirrhosis-associated gastrointestinal infections highlight key technical barriers that could impede industrial progress. Challenges such as biological resistance and regulatory shortcomings require careful attention and examination [56]. Phage therapy faces the four following challenges:

1. Extreme strain-level specificity

The highly specific nature of phage therapy, targeting limited strains, hampers its clinical use because it prevents quick empirical treatment that depends on time-consuming pathogen isolation and accurate in vitro matching. Its narrow host range makes monotherapy ineffective for infections involving multiple microbes, requiring complex multi-phage cocktails that pose significant standardization and regulatory challenges. Additionally, this receptor dependence makes the therapy vulnerable to bacteria developing resistance rapidly through minor phenotypic mutations [138].

2. Rapid Evolution of Resistance and Mutational Pressure

Despite the evolutionary background, the results suggest that high phage doses prompt rapid bacterial adaptation, given that bacteria have evolved complex anti-phage defenses over billions of years. Receptor mutation is the most common initial defense, where bacteria conceal their binding sites to block phage attachment. Therefore, restriction-modification systems and CRISPR-Cas networks have a significant ability to destroy viral nucleic acids. Given these crucial defenses, resistance can develop within days of treatment, leading to a rebound in inflammatory markers and treatment failure.

Recent technological advances, such as engineered phages, phage cocktails, and novel delivery systems, are discussed together with regulatory, manufacturing, and translational barriers. Although engineered and natural phage therapies offer highly promising complementary approaches to addressing the crisis of AMR, their translation into routine clinical practice remains hampered by multiple systemic barriers. Future industrial development must rely on a coordinated regulatory pathway for live-virus therapeutics, GMP-compliant large-scale manufacturing processes, and more rigorously designed randomized controlled clinical trials with larger sample sizes [56].

3. Pharmacokinetic Hurdles: RES Clearance and Immunogenicity

The anatomical and immune landscape in cirrhosis likely presents major challenges for effective phage delivery. Intravenous phages are rapidly cleared from circulation primarily by the reticuloendothelial system, particularly by Kupffer cells in the liver and spleen. Furthermore, the presence of portosystemic shunts alters blood hemodynamics, leading to highly unpredictable effects on phage distribution and half-life [139]. Given that target tissue concentrations consistently appear insufficient, achieving the required multiplicity of infection in target tissues remains critically difficult [140].

Immunogenicity compounds these barriers significantly. Phage capsids are complex foreign proteins, and repeated exposure induces high-affinity neutralizing antibodies that block bacterial infection and accelerate phage clearance [138,140]. Therefore, this creates a critical barrier for long-term chronic treatment of resistant infections. Notwithstanding important recent advances, immunological responses undermine sustained therapeutic efficacy.

Immunity limits chronic treatment viability.

4. Ecological Risks, Manufacturing, and Regulation

Safety remains a critical concern in this study. Genomes must be sequenced to ensure phages are 100% strictly lytic. Given that temperate phages cause iatrogenic super-microbiome disasters, integrating viral DNA into the host microbiota represents a key risk. Additionally, large-scale manufacturing must meet stringent GMP standards. Regulatory frameworks show current rules are too rigid for living drugs. In light of the evidence demonstrating that living drugs require frequent component updates, regulatory frameworks appear unable to adapt to counter bacterial evolution [141].

#### 3.3.6. Critical Analysis

Current research has demonstrated the therapeutic potential of phage therapy for treating microbiota dysbiosis in the gut–liver axis associated with cirrhosis—particularly against pathogenic bacteria such as Enterococcus faecalis and Klebsiella pneumoniae—providing proof of concept for precision antimicrobial interventions. However, this field remains constrained by the disconnect between preclinical models and the complex pathophysiological mechanisms in humans, the lack of direct in vivo causal evidence, and the paradox that large-scale bacterial lysis may trigger endotoxin surges and exacerbate inflammation. Most studies have focused on the isolation and identification of phages and their ecological characterization based on sequencing data, rather than overcoming the complex intestinal barrier, optimizing bactericidal mechanisms through engineering, and conducting standardized clinical validation. To achieve clinical translation, the following key issues must be addressed: establishing direct in vivo causal validation models to elucidate mechanisms, developing engineered phage technologies with low endotoxin release, and deeply integrating phage therapy with gut barrier repair strategies.

## 4. Discussion

### 4.1. The Most Promising Phage Delivery Technologies for the Future

**Figure 3:**
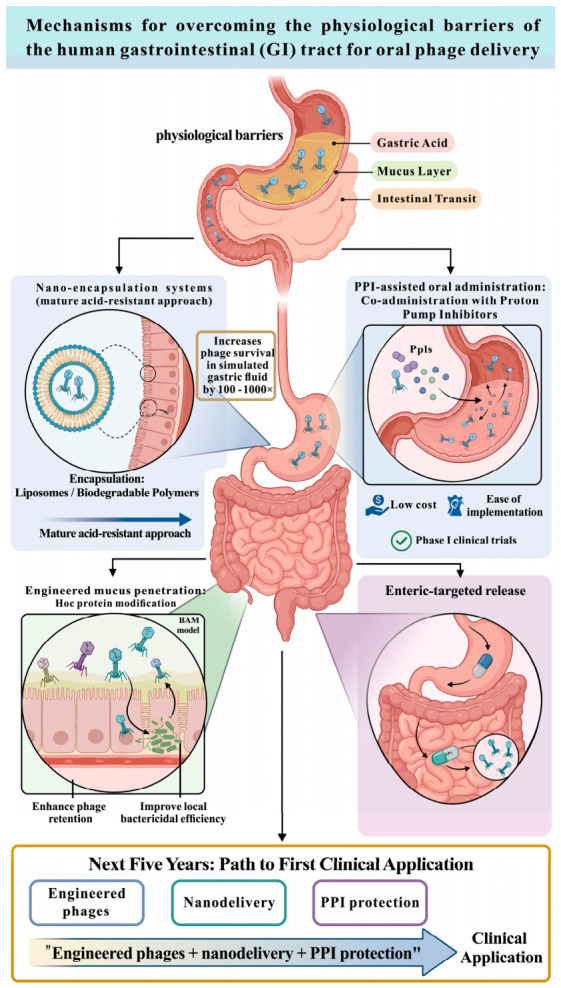
Promising phage delivery technologies for gastrointestinal applications. The schematic illustrates four primary strategies to overcome physiological barriers such as gastric acid and the intestinal mucus layer. These include: (1) Nano-encapsulation systems utilizing liposomes and biodegradable polymers, representing a mature acid-resistant approach to significantly increase phage survival in gastric fluid (by 100–1000×); (2) PPI-assisted oral administration, a low-cost, easily implemented approach utilizing proton pump inhibitors that has already entered Phase I clinical trials; (3) Engineered mucus penetration via Hoc protein modification based on the BAM model to enhance phage retention and improve local bactericidal efficiency; and (4) Enteric-targeted release. Over the next five years, a triple-combination approach integrating engineered phages, nanodelivery, and PPI protection is anticipated to lead the path to the first clinical application.

Overcoming the physiological barriers of the gastrointestinal tract is a core prerequisite for the clinical translation of phages [140]. Currently, the delivery strategies with the greatest translational potential fall into four categories, as shown in Figure 3: nano-encapsulation systems, PPI-assisted oral administration, engineered mucus penetration, and enteric-targeted release. Among these, encapsulation using liposomes and biodegradable polymers can increase phage survival rates in simulated gastric fluid by 100–1000-fold, representing the most mature acid-resistant approach [59,60]; PPI co-administration has entered Phase I clinical trials due to its low cost and ease of implementation [100]; and Hoc protein modification based on the BAM model can enhance phage retention in the mucosal layer, thereby improving local bactericidal efficiency [54,84]. Within the next five years, the triple-combination approach of “engineered phages + nanodelivery + PPI protection” is most likely to be the first to achieve clinical application.

### 4.2. Urgent Improvements Needed at the Policy/Regulatory Level

The widespread clinical adoption of phage therapy is hindered by lagging regulatory frameworks [56,138]. As “living biologics,” their characteristics—including host–range drift, batch-to-batch variability, and dynamic updates—cannot be addressed through traditional regulatory pathways for chemical drugs [141]. Currently, Europe and the United States only permit compassionate use [133,136], and there are no unified GMP production standards or toxicological evaluation guidelines; meanwhile, personalized phage cocktails, which are tailored on a “one patient, one formula” basis, struggle to meet traditional new drug application requirements [56,133]. Future regulation must establish flexible approval pathways, rapid matching platforms, and dynamic licensing updates, while clearly defining mandatory quality control thresholds—such as genomic sequencing, the absence of toxin or resistance genes, and strict lysis-type requirements—to facilitate the transition of phage therapy from research to standardized treatment [57,58].

### 4.3. Concluding Critique

Overall, gastrointestinal phage therapy remains at a stage characterized by “abundant basic research but slow clinical translation.” Most studies remain confined to in vitro and animal models, characterized by small clinical sample sizes, a lack of controls, and single-endpoint designs; simultaneously, there is an overreliance on natural phages, with insufficient investment in engineering and delivery technologies. To truly address drug-resistant infections and dysbiosis, research must shift from phage discovery to systematic studies focused on phage design, delivery optimization, and regulatory standardization [61,62].

## 5. Conclusions

Although phage therapy shows immense potential for gastrointestinal infections, its clinical translation is hindered by host physiological barriers and bacteria-phage co-evolution. The GI tract poses severe biochemical challenges: extreme gastric acidity drastically reduces phage survival [60], and the intestinal mucus layer exhibits a paradoxical “double-edged sword” effect [54].

Furthermore, rapid bacterial adaptation undermines therapeutic efficacy. Bacteria quickly develop resistance using sophisticated defenses like receptor mutations, restriction-modification systems [142], CRISPR-Cas immunity [143,144,145], and prophage-mediated mechanisms [143], leading to a rapid rebound in bacterial populations [142,144]. Additionally, the inadvertent use of temperate phages poses ecological safety risks, as they can facilitate the horizontal transfer of antibiotic resistance genes (ARGs) and virulence factors to the commensal microbiota [145].

Importantly, the current literature primarily establishes correlative—not causal—links between gut phageome alterations and disease. Hypotheses that phages “drive dysbiosis” or “trigger inflammation” still urgently require robust in vivo and clinical validation.

To overcome delivery difficulties and rapid bacterial resistance, future breakthroughs must shift from the “passive screening” of natural phages to “active design.” By adopting a macro-microecological perspective and leveraging cutting-edge computational and engineering technologies, phages can be transformed into intelligent tools for the precise modulation of intestinal microecology.

## Figures and Tables

**Table 1:** Comparison of lytic and temperate bacteriophages.

Feature	Lytic Phages	Temperate Phages	References
Primary Action	Directly lyses and kills the target bacteria.	Integrate into the bacterial genome (prophage).	[5,7]
Life Cycle Stages	Adsorption, penetration, replication, assembly, and release.	Exist in a stable lysogenic state; replicate with the host.	[6,8]
Host Adaptation	Rapidly reduce target population density.	Enhance host fitness, defence, and genetic diversity.	[9,10]
Ecological Model	“Kill-the-Winner” (controls fast-growing populations).	“Piggyback-the-Winner” (coexists with host under stress).	[4,11]
Clinical Safety	High (precision targeting, no collateral damage).	Low (risk of horizontal transfer of resistance genes).	[12,13,14]
Environmental Triggers	Obligate lysis (independent of host stress).	Switch to lysis triggered by DNA damage or oxidative stress.	[15]

**Table 3:** Overview of key phage clinical trials for IBD and intestinal infections.

Sponsor/Research and Development Institution	Phage Candidate Drugs	Targeting Pathogenic Bacteria	Main clinical Indications	Experimental Stage	Clinical Trial Number
BiomX, Inc. (Ness Ziona, Israel).	BX002-A/BX003	K. pneumoniae	IBD/PSC	Phase 1 (Completed)	NCT04737876
Intralytix, Inc. (Columbia, MD, USA).	EcoActive	AIEC	CD	1/2a Phase	NCT03808103
Intralytix, Inc.	VRELysin	Enterococcus	Gastrointestinal VRE colonization clearance	1/2 Phase	NCT05715619
Intralytix, Inc.	Shigella Phage	Shigella	Acute intestinal infection	1/2 Phase	NCT05182749

**Table 4:** Difference between cirrhosis-related and general gastrointestinal infections.

Comparison Dimension	Common Gastrointestinal Infections in Immunocompetent Individuals	Cirrhosis-Associated Gastrointestinal Infections	References
Primary Source & Route of Infection	Exogenous exposure (contaminated food/water), classic fecal-oral transmission	Endogenous outbreak, mainly caused by small intestinal bacterial overgrowth (SIBO) and bacterial translocation induced by gut microbiota dysbiosis	[100,107,108]
Core Pathogens	Specific pathogens: Salmonella, Shigella, Listeria, and gastrointestinal viruses	Opportunistic pathogens: Escherichia coli, Enterococcus faecalis, Klebsiella pneumoniae, often accompanied by overgrowth of fungi (Candida)	[107,109]
Intestinal Barrier Physiological Status	Mucosa undergoes transient inflammatory physical injury; tight junctions are generally intact, and the injury is reversible	Chronic, progressive mechanical and chemical barrier breakdown, leading to severe “leaky gut” syndrome	[100,110,111,112,113]
Hepatic & Systemic Immune Response	Presents as an acute pro-inflammatory self-limiting reaction; pathogens are rapidly recognized and cleared by macrophages and neutrophils	Typical cirrhosis-associated immune dysfunction (CAID); phagocytic activity of the reticuloendothelial system is significantly reduced, accompanied by systemic cytokine storm and immune paralysis	[99,114]
Clinical Outcome & Major Complications	Nausea, vomiting, acute diarrhea, fever, and dehydration; almost no risk of systemic dissemination	Highly prone to progression to systemic infection, directly triggering spontaneous bacterial peritonitis (SBP), hepatic encephalopathy, and acute-on-chronic liver failure (ACLF), with extremely high mortality	[99,102,115]

## Data Availability

No new data were created or analyzed in this study. Data sharing is not applicable to this article.

## Abbreviations

The following abbreviations are used in this manuscript:

ACLF	Acute-on-chronic liver failure
AIEC	Adherent-invasive Escherichia coli
AMR	Antimicrobial resistance
APC	Antigen-presenting cells
ARGs	Antibiotic resistance genes
AUD	Alcohol Use Disorder
BAM	Bacteriophage Adherence to Mucus
BT	Bacterial Translocation
CAID	Cirrhosis-associated immune dysfunction
CARS	Compensatory Anti-Inflammatory Response Syndrome
CD	Crohn’s disease
CDI	Clostridioides difficile infection/Clostridium difficile infection
CRKP	Carbapenem-resistant Klebsiella pneumoniae
DAI	Disease Activity Index
DSS	Dextran sulfate sodium
FMT	Fecal microbiota transplantation
FXR	Farnesoid X Receptor
GALT	Gut-associated lymphoid tissue
GI	Gastrointestinal
GMP	Good Manufacturing Practice
GWAS	Genome-wide association studies
HE	Hepatic Encephalopathy
HGT	Horizontal Gene Transfer
IBD	Inflammatory bowel disease
IFN-γ	Interferon-gamma
ISGs	Interferon-stimulated genes
IV	Intravenous
JAK	Janus kinase
LPS	Lipopolysaccharides (endotoxins)
MDR	Multidrug-resistant
NAFLD	Non-alcoholic fatty liver disease
NK (cells)	Natural killer (cells)
PAMPs	Pathogen-associated molecular patterns
PD	Pharmacodynamics
PK	Pharmacokinetics
PPIs	Proton pump inhibitors
PRRs	Pattern recognition receptors
PTCD	Percutaneous transhepatic cholangial drainage
RCTs	Randomized controlled trials
RES	Reticuloendothelial system
R-M (systems)	Restriction-modification (systems)
ROS	Reactive oxygen species
RUT	Rapid urease test
SBP	Spontaneous bacterial peritonitis
SCFA	Short-chain fatty acid
SDS-PAGE	Sodium dodecyl sulfate-polyacrylamide gel electrophoresis
SIBO	Small intestinal bacterial overgrowth
SIRS	Systemic Inflammatory Response Syndrome
SOC	Standard of care
TLR	Toll-like receptors
UC	Ulcerative colitis
VE-cadherin	Vascular endothelial cadherin
ZO-1	Zonula Occludens-1

## References

[1] Zhang Y., Zhang Y., Chen Z., Jia Z., Yu Y., Wang J., Liang H. (2025). Global Burden, Subtype, Risk Factors and Etiological Analysis of Enteric Infections from 1990-2021: Population Based Study. Front. Cell. Infect. Microbiol..

[2] Dou Z., Zheng H., Shi Y., Li Y., Jia J. (2024). Analysis of Global Prevalence, DALY and Trends of Inflammatory Bowel Disease and Their Correlations with Sociodemographic Index: Data from 1990 to 2019. Autoimmun. Rev..

[3] Qi X., Li Y., Zhu Y., Shen R., Xie Z. (2025). Rebuilding the gut ecosystem: Emerging strategies targeting the microbiota in antibiotic-associated diarrhea. Acta Microbiol. Immunol. Hung..

[4] Duan Y., Young R., Schnabl B. (2022). Bacteriophages and Their Potential for Treatment of Gastrointestinal Diseases. Nat. Rev. Gastroenterol. Hepatol..

[5] Howard-Varona C., Hargreaves K.R., Abedon S.T., Sullivan M.B. (2017). Lysogeny in Nature: Mechanisms, Impact and Ecology of Temperate Phages. ISME J..

[6] Marco R., Jazwinski S.M., Kornberg A. (1974). Binding, Eclipse, and Penetration of the Filamentous Bacteriophage M13 in Intact and Disrupted Cells. Virology.

[7] Liang G., Bushman F.D. (2021). The Human Virome: Assembly, Composition and Host Interactions. Nat. Rev. Microbiol..

[8] Wu M., Zhu Y., Yang Y., Gong Y., Chen Z., Liao B., Xiong Y., Zhou X., Li Y. (2023). SVep1, a Temperate Phage of Human Oral Commensal Streptococcus Vestibularis. Front. Microbiol..

[9] Youssef R.A., Sakr M.M., Shebl R.I., Aboshanab K.M. (2025). Recent Insights on Challenges Encountered with Phage Therapy against Gastrointestinal-Associated Infections. Gut Pathog..

[10] Proença M., Tanoeiro L., Fox J.G., Vale F.F. (2025). Prophage Dynamics in Gastric and Enterohepatic Environments: Unraveling Ecological Barriers and Adaptive Transitions. ISME Commun..

[11] Kurilovich E., Geva-Zatorsky N. (2025). Effects of Bacteriophages on Gut Microbiome Functionality. Gut Microbes.

[12] Lai Z.H., Xie Y.L., Ma C., Feng X.Y., Cao H. (2016). Influence of phages on human intestinal microecological balance and antibiotic resistance. Chin. J. Zoonoses.

[13] Camarillo-Guerrero L.F., Almeida A., Rangel-Pineros G., Finn R.D., Lawley T.D. (2021). Massive Expansion of Human Gut Bacteriophage Diversity. Cell.

[14] Lopez J.A., McKeithen-Mead S., Shi H., Nguyen T.H., Huang K.C., Good B.H. (2025). Abundance Measurements Reveal the Balance between Lysis and Lysogeny in the Human Gut Microbiome. Curr. Biol..

[15] Mäntynen S., Laanto E., Oksanen H.M., Poranen M.M., Díaz-Muñoz S.L. (2021). Black Box of Phage-Bacterium Interactions: Exploring Alternative Phage Infection Strategies. Open Biol..

[16] Thorpe H.M., Wilson S.E., Smith M.C. (2000). Control of Directionality in the Site-Specific Recombination System of the Streptomyces Phage phiC31. Mol. Microbiol..

[17] Bondy-Denomy J., Davidson A.R. (2014). When a Virus Is Not a Parasite: The Beneficial Effects of Prophages on Bacterial Fitness. J. Microbiol..

[18] Quesada J.M., Soriano M.I., Espinosa-Urgel M. (2012). Stability of a Pseudomonas Putida KT2440 Bacteriophage-Carried Genomic Island and Its Impact on Rhizosphere Fitness. Appl. Environ. Microbiol..

[19] Brenes L.R., Laub M.T. (2025). *E. coli*Prophages Encode an Arsenal of Defense Systems to Protect against Temperate Phages. Cell Host Microbe.

[20] Naorem S.S., Han J., Wang S., Lee W.R., Heng X., Miller J.F., Guo H. (2017). DGR Mutagenic Transposition Occurs via Hypermutagenic Reverse Transcription Primed by Nicked Template RNA. Proc. Natl. Acad. Sci. USA.

[21] Liu Z., Yang Y., Mao S., Wang Z., Zhu Q., Yuan Y., Xiang Y. (2025). Extensive Cultivation of Human Gut Phages Revealing Undescribed Bacteroidaceae Phages. Gut Microbes.

[22] Dahlman S., Avellaneda-Franco L., Rutten E.L., Gulliver E.L., Solari S., Chonwerawong M., Kett C., Subedi D., Young R.B., Campbell N. (2025). Isolation, engineering and ecology of temperate phages from the human gut. Nature.

[23] Barr J.J. (2019). Missing a Phage: Unraveling Tripartite Symbioses within the Human Gut. mSystems.

[24] Rothschild-Rodriguez D., Hedges M., Kaplan M., Karav S., Nobrega F.L. (2023). Phage-Encoded Carbohydrate-Interacting Proteins in the Human Gut. Front. Microbiol..

[25] Marantos A., Mitarai N., Sneppen K. (2022). From Kill the Winner to Eliminate the Winner in Open Phage-Bacteria Systems. PLoS Comput. Biol..

[26] Quirós P., Colomer-Lluch M., Martínez-Castillo A., Miró E., Argente M., Jofre J., Navarro F., Muniesa M. (2014). Antibiotic Resistance Genes in the Bacteriophage DNA Fraction of Human Fecal Samples. Antimicrob. Agents Chemother..

[27] Mazaheri Nezhad Fard R., Barton M.D., Heuzenroeder M.W. (2011). Bacteriophage-Mediated Transduction of Antibiotic Resistance in Enterococci. Lett. Appl. Microbiol..

[28] Zhang Y., Guo Y., Qiu T., Gao M., Wang X. (2022). Bacteriophages: Underestimated Vehicles of Antibiotic Resistance Genes in the Soil. Front. Microbiol..

[29] Cadamuro R.D., Elois M.A., Pilati G.V.T., Savi B.P., Pessi L., Jempierre Y.F.S.H., Rodríguez-Lázaro D., Fongaro G. (2025). Role of Lysogenic Phages in the Dissemination of Antibiotic Resistance Genes Applied in the Food Chain. Foods.

[30] Gao H., Wang Y., Zhao Y., Jiao X., Guo Z., Zheng L., Li Y., Su Y., Wang Z., Bai J. (2025). Human Gut Prophage Landscape Identifies a Prophage-Mediated Fucosylation Mechanism Alleviating Colitis. Nat. Commun..

[31] Gogokhia L., Buhrke K., Bell R., Hoffman B., Brown D.G., Hanke-Gogokhia C., Ajami N.J., Wong M.C., Ghazaryan A., Valentine J.F. (2019). Expansion of Bacteriophages Is Linked to Aggravated Intestinal Inflammation and Colitis. Cell Host Microbe.

[32] Majewska J., Kaźmierczak Z., Lahutta K., Lecion D., Szymczak A., Miernikiewicz P., Drapała J., Harhala M., Marek-Bukowiec K., Jędruchniewicz N. (2019). Induction of Phage-Specific Antibodies by Two Therapeutic Staphylococcal Bacteriophages Administered per Os. Front. Immunol..

[33] Babickova J., Gardlik R. (2015). Pathological and Therapeutic Interactions between Bacteriophages, Microbes and the Host in Inflammatory Bowel Disease. World J. Gastroenterol..

[34] Douadi C., Lamy-Besnier Q., Theodorou I., Schiettekatte O., Sbardella Y., Brot L., Costantini P.E., Saporetti R., Danielli A., Calvaresi M. (2026). Differential Translocation of Bacteriophages across the Intestinal Barrier in Health and Crohn’s Disease. Cell Rep..

[35] Kelly C.R., Fischer M., Allegretti J.R., LaPlante K., Stewart D.B., Limketkai B.N., Stollman N.H. (2021). ACG Clinical Guidelines: Prevention, Diagnosis, and Treatment of Clostridioides Difficile Infections. Am. J. Gastroenterol..

[36] Raeisi H., Noori M., Azimirad M., Mohebbi S.R., Asadzadeh Aghdaei H., Yadegar A., Zali M.R. (2023). Emerging Applications of Phage Therapy and Fecal Virome Transplantation for Treatment of Clostridioides Difficile Infection: Challenges and Perspectives. Gut Pathog..

[37] Elbehiry A., Abalkhail A., Anajirih N., Alkhamisi F., Aldamegh M., Alramzi A., AlShaqi R., Alotaibi N., Aljuaid A., Alzahrani H. (2024). Helicobacter Pylori: Routes of Infection, Antimicrobial Resistance, and Alternative Therapies as a Means to Develop Infection Control. Diseases.

[38] Salahi-Niri A., Nabavi-Rad A., Monaghan T.M., Rokkas T., Doulberis M., Sadeghi A., Zali M.R., Yamaoka Y., Tacconelli E., Yadegar A. (2024). Global Prevalence of Helicobacter Pylori Antibiotic Resistance among Children in the World Health Organization Regions between 2000 and 2023: A Systematic Review and Meta-Analysis. BMC Med..

[39] Nale J.Y., Chutia M., Carr P., Hickenbotham P.T., Clokie M.R.J. (2016). “Get in Early”; Biofilm and Wax Moth (*Galleria Mellonella*) Models Reveal New Insights into the Therapeutic Potential of Clostridium Difficile Bacteriophages. Front. Microbiol..

[40] Sabzali S., Pazhouhnia S., Shahzamani K., Sedeh P.A. (2025). Role of Phage Therapy in Acute Gastroenteritis. J. Res. Med. Sci..

[41] Czepiel J., Dróżdż M., Pituch H., Kuijper E.J., Perucki W., Mielimonka A., Goldman S., Wultańska D., Garlicki A., Biesiada G. (2019). Clostridium Difficile Infection: Review. Eur. J. Clin. Microbiol. Infect. Dis..

[42] Northern Ireland Regional Infection Prevention and Control Manual. C difficle. https://www.niinfectioncontrolmanual.net/c-difficle/.

[43] Selle K., Fletcher J.R., Tuson H., Schmitt D.S., McMillan L., Vridhambal G.S., Rivera A.J., Montgomery S.A., Fortier L.-C., Barrangou R. (2020). In Vivo Targeting of Clostridioides Difficile Using Phage-Delivered CRISPR-Cas3 Antimicrobials. mBio.

[44] Myrou A. (2024). Molecular Mechanisms and Treatment Strategies for Helicobacter Pylori-Induced Gastric Carcinogenesis and Mucosa-Associated Lymphoid Tissue (MALT) Lymphoma. Cureus.

[45] Khosravi S., Amini R., Arabestani M.R., Talebi S.S., Jalilian F.A. (2021). Isolation of a Lytic Bacteriophage for *Helicobacter pylori*. Gene Rep..

[46] Hafez R., El-Didamony G., Wagih Abd Elkader E., Said Elazzoni A., Mohammed Basha O., Mohamed A.M., Mohammed H.A. (2026). Anti-Helicobacter Pylori Activity of Egyptian Medicinal Plants and Bacteriophages. ResearchGate.

[47] Schmid E.N., von Recklinghausen G., Ansorg R. (1990). Bacteriophages in Helicobacter (Campylobacter) Pylori. J. Med. Microbiol..

[48] Uchiyama J., Takeuchi H., Kato S., Takemura-Uchiyama I., Ujihara T., Daibata M., Matsuzaki S. (2012). Complete Genome Sequences of Two Helicobacter Pylori Bacteriophages Isolated from Japanese Patients. J. Virol..

[49] Verma V., Devi A., Jhunjhunwala B., Kumar R., Singh J., Pandey M.C., Patwa A., Verma V., Devi A., Jhunjhunwala B. (2025). Evaluation of the Safety and Efficacy of Ganga Water-Phage Therapy Plus Standard Medical Therapy Compared to Standard Medical Therapy Alone for Helicobacter Pylori-Related Dyspepsia: A Randomized Controlled Trial. Cureus.

[50] Ferreira R., Sousa C., Gonçalves R.F.S., Pinheiro A.C., Oleastro M., Wagemans J., Lavigne R., Figueiredo C., Azeredo J., Melo L.D.R. (2022). Characterization and Genomic Analysis of a New Phage Infecting Helicobacter Pylori. Int. J. Mol. Sci..

[51] Nasirov N.G., Sukhorukov B.I., Shabarchina L.I., Petrov A.I. (1991). DNA-protein cross-links as a possible reason for genomic damage during its protonation. Mol. Biol..

[52] Green S.I., Gu Liu C., Yu X., Gibson S., Salmen W., Rajan A., Carter H.E., Clark J.R., Song X., Ramig R.F. (2021). Targeting of Mammalian Glycans Enhances Phage Predation in the Gastrointestinal Tract. mBio.

[53] Barua S., Mitragotri S. (2014). Challenges Associated with Penetration of Nanoparticles across Cell and Tissue Barriers: A Review of Current Status and Future Prospects. Nano Today.

[54] Bhale B., Shirodkar S., Sawarkar S., Omri A. (2026). Phage Therapy: Innovations, Challenges, and Future Directions in Combating Superbugs and Antibiotic Resistance. Expert. Rev. Anti Infect. Ther..

[55] Gummalla V.S., Zhang Y., Liao Y.-T., Wu V.C.H. (2023). The Role of Temperate Phages in Bacterial Pathogenicity. Microorganisms.

[56] Madsen J.S., Burmølle M., Hansen L.H., Sørensen S.J. (2012). The Interconnection between Biofilm Formation and Horizontal Gene Transfer. FEMS Immunol. Med. Microbiol..

[57] Malik D.J., Sokolov I.J., Vinner G.K., Mancuso F., Cinquerrui S., Vladisavljevic G.T., Clokie M.R.J., Garton N.J., Stapley A.G.F., Kirpichnikova A. (2017). Formulation, Stabilisation and Encapsulation of Bacteriophage for Phage Therapy. Adv. Colloid. Interface Sci..

[58] Lorenzo-Rebenaque L., Malik D.J., Catalá-Gregori P., Marin C., Sevilla-Navarro S. (2021). In Vitro and In Vivo Gastrointestinal Survival of Non-Encapsulated and Microencapsulated Salmonella Bacteriophages: Implications for Bacteriophage Therapy in Poultry. Pharmaceuticals.

[59] King S.H., Driscoll C.L., Li D.B., Guo D., Merchant A.T., Brixi G., Wilkinson M.E., Hie B.L. (2025). Generative Design of Novel Bacteriophages with Genome Language Models. bioRxiv.

[60] Lood C., Boeckaerts D., Stock M., De Baets B., Lavigne R., van Noort V., Briers Y. (2022). Digital Phagograms: Predicting Phage Infectivity through a Multilayer Machine Learning Approach. Curr. Opin. Virol..

[61] Li J., Li Y., Ding Y., Huang C., Zhang Y., Wang J., Wang X. (2021). Characterization of a Novel Siphoviridae Salmonella Bacteriophage T156 and Its Microencapsulation Application in Food Matrix. Food Res. Int..

[62] Kim E.-J., Ryu S., Lim J.-A. (2025). Bacteriophage Cocktail LEC2-LEC10 for Broad-Spectrum Control of Pathogenic and Uncharacterized Escherichia Coli in Fresh Produce. Front. Microbiol..

[63] Lin L.-C., Tsai Y.-C., Lin N.-T. (2025). Phage-Antibiotic Synergy Enhances Biofilm Eradication and Survival in a Zebrafish Model of Pseudomonas Aeruginosa Infection. Int. J. Mol. Sci..

[64] Kutateladze M., Adamia R. (2008). Phage Therapy Experience at the Eliava Institute. Médecine Mal. Infect..

[65] Sarker S.A., McCallin S., Barretto C., Berger B., Pittet A.-C., Sultana S., Krause L., Huq S., Bibiloni R., Bruttin A. (2012). Oral T4-like Phage Cocktail Application to Healthy Adult Volunteers from Bangladesh. Virology.

[66] Sarker S.A., Sultana S., Reuteler G., Moine D., Descombes P., Charton F., Bourdin G., McCallin S., Ngom-Bru C., Neville T. (2016). Oral Phage Therapy of Acute Bacterial Diarrhea With Two Coliphage Preparations: A Randomized Trial in Children From Bangladesh. EBioMedicine.

[67] Han T., Zhang Y., Zheng G., Guo Y. (2025). From Pathogenic Mechanisms to Therapeutic Perspectives: A Review of Gut Microbiota and Intestinal Mucosal Immunity in Inflammatory Bowel Disease. Front. Immunol..

[68] Diez-Martin E., Hernandez-Suarez L., Muñoz-Villafranca C., Martin-Souto L., Astigarraga E., Ramirez-Garcia A., Barreda-Gómez G. (2024). Inflammatory Bowel Disease: A Comprehensive Analysis of Molecular Bases, Predictive Biomarkers, Diagnostic Methods, and Therapeutic Options. Int. J. Mol. Sci..

[69] Yang X., Guo H., Zou M. (2026). Inflammatory Bowel Diseases: Pathological Mechanisms and Therapeutic Perspectives. Mol. Biomed..

[70] Vestergaard M.V., Alfaro-Núñez A., Sazonovs A., Athanasiadis G., Jess T. (2026). Multimodal Analysis Disentangles the Genetic and Microbial Associations between Inflammatory Bowel Disease and Other Immune-Mediated Diseases across a Harmonized Population Framework. Nat. Commun..

[71] Kou R., Guo Y., Qin Z., Xu X., Liu Y., Wei W., Chen Y., Jian Z., Lan B. (2025). Systemic Dysregulation of the Gut Microenvironment Plays a Pivotal Role in the Onset and Progression of Inflammatory Bowel Disease. Front. Immunol..

[72] Calvez V., Puca P., Di Vincenzo F., Del Gaudio A., Bartocci B., Murgiano M., Iaccarino J., Parand E., Napolitano D., Pugliese D. (2025). Novel Insights into the Pathogenesis of Inflammatory Bowel Diseases. Biomedicines.

[73] Shen X., Liu A., Liang X., Zhang X. (2024). Phage: Future Treatment Direction for Inflammatory Bowel Disease Patients. Microbiota Dis..

[74] Feng Z., Burgermeister E., Philips A., Zuo T., Wen W. (2025). The Gut Virome in Association with the Bacteriome in Gastrointestinal Diseases and beyond: Roles, Mechanisms, and Clinical Applications. Prec Clin. Med..

[75] Tun H.M., Peng Y., Massimino L., Sin Z.Y., Parigi T.L., Facoetti A., Rahman S., Danese S., Ungaro F. (2024). Gut Virome in Inflammatory Bowel Disease and Beyond. Gut.

[76] Gogokhia L., Round J.L. (2021). Immune-Bacteriophage Interactions in Inflammatory Bowel Diseases. Curr. Opin. Virol..

[77] Ding B., Fan M., Shi Y.-P., Chen X., Duan Y. (2025). Mechanistic Roles and Therapeutic Potential of Bacteriophages in Inflammatory Gastrointestinal Diseases. Microbiome Res. Rep..

[78] Rahaman M.M., Wangchuk P., Sarker S. (2025). Targeting Interferon-Gamma (IFN-γ)-Related Signalling Pathways in Inflammatory Bowel Disease: Emerging Inhibitors and Therapeutic Advances. Mediat. Inflamm..

[79] Hu J., Ye H., Wang S., Wang J., Han D. (2021). Prophage Activation in the Intestine: Insights Into Functions and Possible Applications. Front. Microbiol..

[80] Wu J., Fu K., Hou C., Wang Y., Ji C., Xue F., Ren J., Dai J., Barr J.J., Tang F. (2024). Bacteriophage Defends Murine Gut from Escherichia Coli Invasion via Mucosal Adherence. Nat. Commun..

[81] Mahmud M.R., Tamanna S.K., Akter S., Mazumder L., Akter S., Hasan M.R., Acharjee M., Esti I.Z., Islam M.S., Shihab M.M.R. (2024). Role of Bacteriophages in Shaping Gut Microbial Community. Gut Microbes.

[82] Almeida G.M.F., Laanto E., Ashrafi R., Sundberg L.-R. (2019). Bacteriophage Adherence to Mucus Mediates Preventive Protection against Pathogenic Bacteria. mBio.

[83] Daryani N.E., Jazayeri S.M., Izadi N., Ahmadi H., Baghi H.B., Shirmohammadi M., Sabbaghian M., Shekarchi A.A., Marvi S.S., Azadi A. (2026). Characterizing the Gut Virome in Ulcerative Colitis and Crohn’s Disease: Signatures of Disease Severity. Virol. J..

[84] Ayele H., Jo J., Begum K., Hu C., Le T.M., Alam M.J., Eubank T.A., Haidacher S.J., Horvath T.D., Hanson B.M. (2025). A Randomized Phase 1 Study Investigating Gut Microbiome Changes With Moxifloxacin vs Oral Vancomycin: Implications for Clostridioides Difficile Risk. J. Infect. Dis..

[85] Wolfe T.M., Jo J., Pinkham N.V., Garey K.W., Walk S.T. (2025). The Impact of Ibezapolstat and Other Clostridioides Difficile Infection-Relevant Antibiotics on the Microbiome of Humanized Mice. Antimicrob. Agents Chemother..

[86] Umansky A.A., Fortier L.C. (2023). The Long and Sinuous Road to Phage-Based Therapy of Clostridioides Difficile Infections. Front. Med..

[87] Tanaka T., Sugiyama R., Sato Y., Kawaguchi M., Honda K., Iwaki H., Okano K. (2024). Precise Microbiome Engineering Using Natural and Synthetic Bacteriophages Targeting an Artificial Bacterial Consortium. Front. Microbiol..

[88] Easwaran M., Abdelrahman F., El-Shibiny A., Venkidasamy B., Thiruvengadam M., Sivalingam P., Ganapathy D., Ahn J., Shin H.J. (2025). Exploring Bacteriophages to Combat Gut Dysbiosis: A Promising New Frontier in Microbiome Therapy. Microb. Pathog..

[89] Yang C., Merlin D. (2024). Unveiling Colitis: A Journey through the Dextran Sodium Sulfate-Induced Model. Inflamm. Bowel Dis..

[90] Katsandegwaza B., Horsnell W., Smith K. (2022). Inflammatory Bowel Disease: A Review of Pre-Clinical Murine Models of Human Disease. Int. J. Mol. Sci..

[91] Xu J., Liu T., Shao Y., Liu Q., Zhang Z., Yuan Y., Zhang S., Wang Y., Sun L., Zhou S. (2024). Phage Cocktail Inhibits Inflammation and Protects the Integrity of the Intestinal Barrier in Dextran Sulfate Sodium-Induced Colitis Mice Model. Microb. Pathog..

[92] Xie Z., Lv X., Zhong C., Wang F., Zhang Y., Li Y., Huang Y., Yang S., Shi Y. (2024). Protective Effect of Phage pSal-4 on Chicken Intestinal Epithelial Cells Injured by Salmonella Enteritidis. BMC Microbiol..

[93] Islam M.S., Wei P., Zhang K., Nime I., Pan F. (2025). Phage Therapy Modulates the Gut Microbiome and Immune Responses in Non-Typhoidal *Salmonella*-Induced Colitis. Food Res. Int..

[94] Iaquinto G., Aufiero V.R., Mazzarella G., Lucariello A., Panico L., Melina R., Iaquinto S., Luca A.D., Sellitto C. (2024). Pathogens in Crohn’s Disease: The Role of Adherent Invasive *Escherichia coli*. CRE.

[95] Barnich N., Darfeuille-Michaud A. (2007). Adherent-Invasive Escherichia Coli and Crohn’s Disease. Curr. Opin. Gastroenterol..

[96] Galtier M., de Sordi L., Sivignon A., de Vallée A., Neut C., Rahmouni O., Wannerberger K., Darfeuille-Michaud A., Desreumaux P., Barnich N. (2017). Bacteriophages Targeting Adherent Invasive Escherichia Coli Strains as a Promising New Treatment for Crohn’s Disease. J. Crohn’s Colitis.

[97] Jackson K., Galipeau H., Hann A., Constante M., Zangara M., Bording-Jorgensen M., Fuentes A., Ho H., Wang J., Shimbori C. (2025). Phage Intervention Improves Colitis and Response to Corticosteroids by Attenuating Virulence of Crohn’s Disease-Associated Bacteria. bioRxiv.

[98] Zhu J., Liu Y., Chen T., Feng F., Fang T., Zhang W., Li Y., Ju Y., Xu L., Zhuge X. (2025). “Precision-Guided Killer” Engineered Phage for Combating Carbapenem-Resistant Klebsiella Pneumoniae Induced Inflammatory Bowel Disease. Adv. Funct. Mater..

[99] Lockwood C., dos Santos K.B., Pap R. (2019). Practical Guidance for Knowledge Synthesis: Scoping Review Methods. Asian Nurs. Res..

[100] Federici S., Kredo-Russo S., Valdés-Mas R., Kviatcovsky D., Weinstock E., Matiuhin Y., Silberberg Y., Atarashi K., Furuichi M., Oka A. (2022). Targeted Suppression of Human IBD-Associated Gut Microbiota Commensals by Phage Consortia for Treatment of Intestinal Inflammation. Cell.

[101] Lerminiaux N.A., Cameron A.D.S. (2019). Horizontal Transfer of Antibiotic Resistance Genes in Clinical Environments. Can. J. Microbiol..

[102] Davies J., Davies D. (2010). Origins and Evolution of Antibiotic Resistance. Microbiol. Mol. Biol. Rev..

[103] Munita J.M., Arias C.A. (2016). Mechanisms of Antibiotic Resistance. Microbiol. Spectr..

[104] Zhong L., Xu D., He J., Sun L., Fan G., Zhu T., Yao Y., Feng T., Cui Z. (2025). Pre-Exposure to Phage Particles Reduces Their Antibacterial Therapeutic Efficacy Both in Vitro and in Vivo. Int. J. Med. Microbiol..

[105] Gordillo Altamirano F.L., Barr J.J. (2019). Phage Therapy in the Postantibiotic Era. Clin. Microbiol. Rev..

[106] Jalan R., Fernandez J., Wiest R., Schnabl B., Moreau R., Angeli P., Stadlbauer V., Gustot T., Bernardi M., Canton R. (2014). Bacterial Infections in Cirrhosis: A Position Statement Based on the EASL Special Conference 2013. J. Hepatol..

[107] Wiest R., Lawson M., Geuking M. (2014). Pathological Bacterial Translocation in Liver Cirrhosis. J. Hepatol..

[108] Albillos A., Lario M., Álvarez-Mon M. (2014). Cirrhosis-Associated Immune Dysfunction: Distinctive Features and Clinical Relevance. J. Hepatol..

[109] Bajaj J.S., Ng S.C., Schnabl B. (2022). Promises of Microbiome-Based Therapies. J. Hepatol..

[110] Van der Merwe S., Chokshi S., Bernsmeier C., Albillos A. (2021). The Multifactorial Mechanisms of Bacterial Infection in Decompensated Cirrhosis. J. Hepatol..

[111] Bonnel A.R., Bunchorntavakul C., Reddy K.R. (2011). Immune Dysfunction and Infections in Patients With Cirrhosis. Clin. Gastroenterol. Hepatol..

[112] London K.C. Shawcross Lab—Liver-Intestinal-Microbiome-Brain Interactions in Cirrhosis (LiMBIC). https://www.kcl.ac.uk/research/shawcross-lab.

[113] Hsu C., Duan Y., Fouts D.E., Schnabl B. (2021). Intestinal Virome and Therapeutic Potential of Bacteriophages in Liver Disease. J. Hepatol..

[114] Bajaj J.S., Heuman D.M., Hylemon P.B., Sanyal A.J., White M.B., Monteith P., Noble N.A., Unser A.B., Daita K., Fisher A.R. (2014). The Cirrhosis Dysbiosis Ratio Defines Changes in the Gut Microbiome Associated with Cirrhosis and Its Complications. J. Hepatol..

[115] Kakiyama G., Pandak W.M., Gillevet P.M., Hylemon P.B., Heuman D.M., Daita K., Takei H., Muto A., Nittono H., Ridlon J.M. (2013). Modulation of the Fecal Bile Acid Profile by Gut Microbiota in Cirrhosis. J. Hepatol..

[116] Bajaj J.S., Liu E.J., Kheradman R., Fagan A., Heuman D.M., White M., Gavis E.A., Hylemon P., Sikaroodi M., Gillevet P.M. (2018). Fungal Dysbiosis in Cirrhosis. Gut.

[117] Pearson C., Uhlig H.H., Powrie F. (2012). Lymphoid Microenvironments and Innate Lymphoid Cells in the Gut. Trends Immunol..

[118] Spadoni I., Zagato E., Bertocchi A., Paolinelli R., Hot E., Di Sabatino A., Mortha A., Chieppa M., Lumb L.N., Hardwick J.C. (2015). A gut-vascular barrier controls the systemic dissemination of bacteria. Science.

[119] Bellot P., Francés R., Such J. (2013). Pathological Bacterial Translocation in Cirrhosis: Pathophysiology, Diagnosis and Clinical Implications. Liver Int..

[120] Assimakopoulos S.F., Tsamandas A.C., Tsiaoussis G.I., Karatza E., Triantos C., Vagianos C.E., Spiliopoulou I., Kaltezioti V., Charonis A., Nikolopoulou V.N. (2012). Altered Intestinal Tight Junctions’ Expression in Patients with Liver Cirrhosis: A Pathogenetic Mechanism of Intestinal Hyperpermeability. Eur. J. Clin. Investig..

[121] Trebicka J., Fernandez J., Papp M., Caraceni P., Laleman W., Gambino C., Giovo I., Uschner F.E., Jimenez C., Mookerjee R. (2020). The PREDICT Study Uncovers Three Clinical Courses of Acutely Decompensated Cirrhosis That Have Distinct Pathophysiology. J. Hepatol..

[122] Vural A., Kehrl J.H. (2014). Autophagy in Macrophages: Impacting Inflammation and Bacterial Infection. Scientifica.

[123] Kumar V. (2019). Inflammation Research Sails through the Sea of Immunology to Reach Immunometabolism. Int. Immunopharmacol..

[124] Glass E., Robinson S.L., Rosowski E.E. (2025). Zebrafish Use Conserved CLR and TLR Signaling Pathways to Respond to Fungal PAMPs in Zymosan. Dev. Comp. Immunol..

[125] Meng E.X., Verne G.N., Zhou Q. (2024). Macrophages and Gut Barrier Function: Guardians of Gastrointestinal Health in Post-Inflammatory and Post-Infection Responses. Int. J. Mol. Sci..

[126] Dirchwolf M., Ruf A.E. (2015). Role of Systemic Inflammation in Cirrhosis: From Pathogenesis to Prognosis. World J. Hepatol..

[127] Duan Y., Llorente C., Lang S., Brandl K., Chu H., Jiang L., White R.C., Clarke T.H., Nguyen K., Torralba M. (2019). Bacteriophage Targeting of Gut Bacterium Attenuates Alcoholic Liver Disease. Nature.

[128] Demir M., Lang S., Hartmann P., Duan Y., Martin A., Miyamoto Y., Bondareva M., Zhang X., Wang Y., Kasper P. (2022). The Fecal Mycobiome in Non-Alcoholic Fatty Liver Disease. J. Hepatol..

[129] Hsu B.B., Gibson T.E., Yeliseyev V., Liu Q., Lyon L., Bry L., Silver P.A., Gerber G.K. (2019). Dynamic Modulation of the Gut Microbiota and Metabolome by Bacteriophages in a Mouse Model. Cell Host Microbe.

[130] Thingstad T.F. (2000). Elements of a Theory for the Mechanisms Controlling Abundance, Diversity, and Biogeochemical Role of Lytic Bacterial Viruses in Aquatic Systems. Limnol. Oceanogr..

[131] Koskella B., Brockhurst M.A. (2014). Bacteria–Phage Coevolution as a Driver of Ecological and Evolutionary Processes in Microbial Communities. FEMS Microbiol. Rev..

[132] Lin D.M., Koskella B., Lin H.C. (2017). Phage Therapy: An Alternative to Antibiotics in the Age of Multi-Drug Resistance. World J. Gastrointest. Pharmacol. Ther..

[133] Schooley R.T., Biswas B., Gill J.J., Hernandez-Morales A., Lancaster J., Lessor L., Barr J.J., Reed S.L., Rohwer F., Benler S. (2017). Development and Use of Personalized Bacteriophage-Based Therapeutic Cocktails To Treat a Patient with a Disseminated Resistant Acinetobacter Baumannii Infection. Antimicrob. Agents Chemother..

[134] Albillos A., de Gottardi A., Rescigno M. (2020). The Gut-Liver Axis in Liver Disease: Pathophysiological Basis for Therapy. J. Hepatol..

[135] Yuan J., Chen C., Cui J., Lu J., Yan C., Wei X., Zhao X., Li N., Li S., Xue G. (2019). Fatty Liver Disease Caused by High-Alcohol-Producing *Klebsiella pneumoniae*. Cell Metab..

[136] Dedrick R.M., Smith B.E., Cristinziano M., Freeman K.G., Jacobs-Sera D., Belessis Y., Whitney Brown A., Cohen K.A., Davidson R.M., van Duin D. (2023). Phage Therapy of Mycobacterium Infections: Compassionate Use of Phages in 20 Patients With Drug-Resistant Mycobacterial Disease. Clin. Infect. Dis..

[137] Febvre H.P., Rao S., Gindin M., Goodwin N.D.M., Finer E., Vivanco J.S., Lu S., Manter D.K., Wallace T.C., Weir T.L. (2019). PHAGE Study: Effects of Supplemental Bacteriophage Intake on Inflammation and Gut Microbiota in Healthy Adults. Nutrients.

[138] Gallina A., Gallina M., Cona A., Vitulo P., Mularoni A., Provenzani A. (2026). Phage Therapy at the Crossroads Between Clinical Promise and Regulatory Challenge. Pharmaceuticals.

[139] Van Espen L., Brol M.J., Close L., Schierwagen R., Gu W., Keller M.I., Balogh B., Fullam A., De Coninck L., Nakamura T. (2026). Lactococcus A Phages Predict ACLF While Enterococcus B Phages Predict Bacterial Infection in Decompensated Cirrhosis. JHEP Rep..

[140] Wacnik M., Hauza E., Skaradzińska A., Śliwka P. (2026). Bacteriophage Therapy: Overcoming Antimicrobial Resistance Through Advanced Delivery Methods. Molecules.

[141] Proposed GMP Exemption for Certain Bacteriophage Manufacture—Therapeutic Goods Administration—Citizen Space. https://consultations.tga.gov.au/medicines-regulation-division/proposed-gmp-exemption-for-phage-manufacture/.

[142] Malone L.M., Birkholz N., Fineran P.C. (2021). Conquering CRISPR: How Phages Overcome Bacterial Adaptive Immunity. Curr. Opin. Biotechnol..

[143] Broniewski J.M., Chisnall M.A.W., Høyland-Kroghsbo N.M., Buckling A., Westra E.R. (2021). The Effect of Quorum Sensing Inhibitors on the Evolution of CRISPR-Based Phage Immunity in Pseudomonas Aeruginosa. ISME J..

[144] Dimitriu T., Kurilovich E., Łapińska U., Severinov K., Pagliara S., Szczelkun M.D., Westra E.R. (2022). Bacteriostatic Antibiotics Promote CRISPR-Cas Adaptive Immunity by Enabling Increased Spacer Acquisition. Cell Host Microbe.

[145] Watson B.N.J., Steens J.A., Staals R.H.J., Westra E.R., van Houte S. (2021). Coevolution between Bacterial CRISPR-Cas Systems and Their Bacteriophages. Cell Host Microbe.

